# Hyperglycemia in combination with excess fat intake promotes renal pyroptosis and fibrosis through Gα_12_-dependent endoplasmic reticulum stress

**DOI:** 10.7150/thno.124015

**Published:** 2026-01-14

**Authors:** Muhammad Sohaib Khan, Boram Kim, Yerim Jeon, Jihoon Tak, Yun Seok Kim, Sang Gil Lee, Eun Byul Lee, Chang-hoon Lee, Cheol Bin Eom, Hyun Sook Lee, Hyeon-Ki Jang, Nakyeom Lee, Jeong Hae Kie, Jee Myung Yang, Yoon Mee Yang, Sang Geon Kim

**Affiliations:** 1 College of Pharmacy, Dongguk University, Goyang-si, Kyeonggi-do 10326, Republic of Korea.; 2 Department of Medicine, Graduate School, Dongguk University, Goyang-si, Kyeonggi-do 10326, Republic of Korea.; 3 Dongguk University Medical Center, Goyang-si, Kyeonggi-do 10326, Republic of Korea.; 4 BK21 FOUR Team and Integrated Research Institute for Drug Development, College of Pharmacy, Dongguk University, Seoul 04620, Republic of Korea.; 5 Department of Anesthesiology, Critical Care and Pain Medicine, McGovern Medical School, The University of Texas Health Science Center at Houston, Houston, TX, 77030, USA.; 6 College of Pharmacy and Research Institute of Pharmaceutical Sciences, Seoul National University, Korea.; 7 Center of Research and Development, A Pharma Inc, Goyang-si, Gyeonggi-do, Republic of Korea.; 8 Department of Ophthalmology, Asan Medical Center, University of Ulsan College of Medicine, #88, Olympic-ro 43-gil, Songpa-gu, Seoul 05505, Republic of Korea.; 9 Kangwon National University, Chuncheon 24341, South Korea.; 10 Multidimensional Genomics Research Center, Kangwon University, Chuncheon 24341, South Korea.; 11 Arlico Pharm, Central Lab, B-dong, #401~9, 338, Gwanggyojungang-ro, Suji-gu, Yongin-si, Gyeonggi-do, Korea.; 12 Department of Pathology, NHIS Ilsan Hospital, Goyang, Gyeonggi-do, 10444, Republic of Korea.; 13 School of Pharmacy, Sungkyunkwan University, Suwon, Gyeonggi-do, 06419, Republic of Korea.

**Keywords:** renal pyroptosis, fibrosis, free fatty acids, hyperglycemia, ER stress, human kidney

## Abstract

**Background:** Chronic exposure to free fatty acids (FFAs) and glucose may disrupt metabolic homeostasis and initiate pathological processes. This study investigated the effects of hyperglycemia and fat overload on renal endoplasmic reticulum (ER) stress, pyroptosis and fibrogenesis in mice and the underlying basis. We hypothesized that the combined insult would more severely induce Gα_12_-dependent ER stress and renal complications.

**Methods:** Mice were subjected to either high fat diet (HFD)+streptozotocin (STZ), or STZ treatment, and AZ2 was used as an anti-diabetic agent. Blood sera were used for blood biochemistry, and tissues were employed for RNA sequencing, immunoblottings, TEM, histology and immunohistochemistry. HEK293 and other cells were used for high glucose (HG) and palmitate treatment, or Gα_12_ or siGα_12_ transfection.

**Results:** The combined HFD and STZ treatment, showing enrichment of genes related to GPCR signaling, inflammasome, ER stress, and pyroptosis in the RNA-sequencing analysis, upregulated Gα_12_ in the kidney, alongside increased PGC1α and PPARα. IRE1α and ATF6 were elevated without an increase in GRP78. This was accompanied by elevated blood glucose, creatinine, and BUN levels. We also found increases of pro-IL-1β, IL-1β, caspase-1, and NLRP3, demonstrating pyroptosis. Immunoassays revealed increased fibrosis markers. AZ2 reversed these changes. STZ treatment alone exhibited mild complications in the absence of Gα_12_ induction despite severe hyperglycemia. In cell-based assays, HG+palmitate elicited IRE1 activation along with Gα_12_ overexpression although HG alone had a minimal effect. Overexpression of Gα_12_ facilitated the effect of HG+palmitate on ER stress, pyroptosis, and fibrosis, whereas Gα_12_ knockdown had the opposite effect, as corroborated by the outcomes obtained using STZ-treated Gα_12_-/-, Gα_12_+/-, and Gα_13_ liver-specific KO mice.

**Conclusion:** These findings support the role of HG and lipid overload combination in driving renal pyroptosis and fibrogenesis through Gα_12_-mediated ER stress and inflammasome, delineating the mechanism underlying the conditions of diabetic renal complications and pharmacological intervention.

## Introduction

Kidneys are vulnerable to damage from diabetes. In diabetic patients, sustained hyperglycemia often impairs kidney function, leading to renal complications 1. While the damaging effects of high blood glucose are well-established, a series of evidence shows the role of lipids in this process 2. However, the combinatorial effect of hyperglycemia and fat overload on diabetic renal complications and the underlying mechanisms remains incompletely understood. This study aims to elucidate the complex interplay between high glucose (HG) and fat, investigating their contribution to catastrophic endoplasmic reticulum (ER) stress. Specifically, we hypothesized that the combined effects of hyperglycemia and fat overload would amplify renal cell pyroptosis, and fibrosis as mediated by the activation of Gα_12_-dependent ER stress and inflammasome activation.

Persistent hyperglycemia initiates morphological and biochemical changes of the cells within the kidney. Increased glucose flux activates detrimental pathways (e.g., polyol and glycation end products) 3. These pathways would induce oxidative stress, inflammation, and extracellular matrix (ECM) accumulation, impairing renal cells. Moreover, hyperglycemia may be accompanied by elevated circulating free fatty acids (FFAs) due to overloaded fat particularly in type 2 diabetes, creating a combined insult that may potentiate renal damage and repair processes 4, 5. This study seeks to determine the extent to which this combined exposure promotes lipotoxicity and additionally finds a pharmacological way of intervention.

A key event of cellular stress in response to hyperglycemia is the ER stress, initiating the unfolded protein response (UPR), aiming to restore ER homeostasis. However, different conditions of chronic ER stress, in the context of combined hyperglycemia and excess fat, may trigger cell death pathways overriding UPR responses 6. Gα_12/13_, members of the G protein family, have been implicated in various processes, including cell growth, differentiation, and inflammation. A line of our studies has shown that Gα_12_ overexpression by ER stress facilitates ferroptosis in hepatocytes through ROCK1 7, and that hepatic injury prompted by ER stress leads to the suppression of NEMO, facilitating ferroptosis 8. In addition, chemical-induced colitis may cause ER stress-induced pyroptosis as mediated by Gα_12_ overexpression 9. In another study, we examined the effect of Gα_12_ overexpression on the activation of hepatic stellate cells, which supports the impact of the Gα_12_ signaling axis on liver fibrosis 10. Given the link of pathologically overexpressed Gα_12_ with different cell death processes and fibrogenesis, the present study investigated the activation of inositol-requiring enzyme 1α (IRE1α), and its association with the induction of Gα_12_, in exacerbating renal complications during chronic diabetes, and if so, what would be the causative factor. We hypothesize that hyperglycemia and fat overload synergistically upregulate the expression of Gα_12_ and thereby induce catastrophic ER stress, leading to inflammasome activation and the resultant renal complications.

Pyroptosis, a form of inflammatory cell death, emerged as a contributor to renal injury in diabetic nephropathy. This study assessed whether combined exposure to hyperglycemia and lipid triggers pyroptosis, leading to the release of pro-inflammatory cytokines, such as IL-1β, and subsequent injury of the renal cells. Our findings revealed increases in IL-1β, caspase-1, and NLR family pyrin domain containing 3 (NLRP3) levels in the kidneys of high fat diet (HFD)+streptozotocin (STZ)-treated mice. Moreover, we try to understand the resultant effects of renal cell death on ECM proteins, which would lead to renal fibrosis. To antagonize the detrimental effect of hyperglycemia and fat overload, we examined the potential of AZ2, an anti-hyperglycemic candidate, to attenuate renal damage in the hyperglycemic mouse model with fat overload. Our results showed that treatment of AZ2 (a functional Gα_12_ inhibitor) reversed the changes induced by HFD+STZ, including the overexpression of Gα_12_, ER stress, pyroptosis, and fibrosis.

This study also examined ER stress markers and Gα_12_ levels in the kidney of mice subjected to STZ treatment alone, and its associations with the extent of renal complications. When we comparatively evaluated the effect of persistent and higher degrees of hyperglycemia induced by STZ, we surprisingly found mild renal injury and complications in the absence of Gα_12_ overexpression and inflammasome-mediated pyroptosis. This concept was verified using cell-based assays employing HG and/or palmitate with or without Gα_12_ modulation, and was further strengthened by the *in vivo* results from genetic knockout (KO) mice experiments. Overall, the outcomes of this study provide an understanding of the driving factor by which hyperglycemia synergistically elicits renal complications during diabetes and the underlying molecular basis.

## Materials and Methods

### Materials

Primary antibodies were sourced from various suppliers as follows: α-SMA (ab7817) was obtained from Abcam (Cambridge, MA, USA); Fibronectin (610078) from BD Biosciences (Franklin Lakes, NJ, USA); and activating transcription factor 6 (ATF6) (NBD1-40256) from Novus Biologicals (Littleton, CO, USA). Sigma-Aldrich (St. Louis, MO, USA) provided the β-actin antibody (A5441), while PGC1α (66369-1-Ig) was procured from Proteintech (Rosemont, IL, USA). Antibodies targeting N-cadherin (13A9), IL-1β (12242), and NLPR3 (15101) were supplied by Cell Signaling Technology (Danvers, MA, USA). A range of antibodies, including Caspase-1 (sc-56036), Col1a (sc-59772), Gα_12_ (sc-515445), Gα_13_ (sc-293424), IRE1α (sc-390960), PPARα (sc-9000), and vimentin (sc-32322), were purchased from Santa Cruz Biotechnology (Dallas, TX, USA). Secondary detection was performed using HRP-conjugated goat anti-rabbit (G-21234) and goat anti-mouse (G-21040) IgGs from Invitrogen (Carlsbad, CA, USA). The AZ2 compound was a generous gift from A Pharma Co. (Goyang, South Korea).

### Human samples

The study used paraffin-embedded kidney tissues obtained from 4 normal subjects and 10 patients with diabetic kidney disease at NHIS Ilsan Hospital (Goyang, Korea). All specimens were reviewed by pathologists, and only deidentified samples were used for analysis. Written informed consent was obtained from all participating patients. The study protocol was approved by the Institutional Review Board (IRB No. DUIRB2025-10-05(F)). Five representative results were shown in each group.

### Diabetic animal models

Animal experiments were carried out at Dongguk University, with ethical approval granted by the Institutional Animal Care and Use Committee (IACUC) under protocols IACUC-2021-040-1 and IACUC-2022-045-1. To study diabetes induction, nine-week-old male wild-type C57BL/6 mice were used. The animals were housed in a regulated environment with a 12 h light/dark cycle, a temperature maintained at 25 ± 2°C, and humidity levels ranging from 50% to 55%. All mice had *ad libitum* access to standard laboratory chow and tap water. Prior to the start of the experimental protocols, the rodents were allowed to acclimatize to the laboratory facility for a period of two weeks.

Animal experiments were conducted in 4 different models. The experiment #1 involved three groups: a normal diet (ND) group (n=5), a HFD plus STZ-treated group (n=4), and an HFD+STZ group treated with AZ2 (n=7). Mice in the HFD groups were allowed free to access a diet containing 60 kcal% fat (D12492, Research Diets, USA) for 18 weeks. At 12^th^ week, the HFD+STZ groups received intraperitoneal injections of STZ (50 mg/kg, dissolved in citrate buffer with pH 4.7) for three consecutive days. The AZ2 was prepared by dissolving in 0.5% carboxymethyl cellulose, was administered orally (p.o.) at a dose of 10 mg/kg, five times a week for a period of three weeks prior to euthanasia. In experiment #2: n = 4 (ND), n = 8 (HFD+STZ), and n = 7 (AZ2), the male mice were fed with HFD for 18 weeks, and were subjected to intraperitoneal STZ injections prepared in citrate buffer solvent (50 mg/kg body weight) for consecutive 3 days in 12^th^ week. Mice were treated with AZ2 30 mg/kg body weight p.o., for 3 weeks.

In another experimental set #3, we employed a STZ model of 15 weeks in 9 weeks old male mice. The mice were divided into two groups: a vehicle (VEH) group (n=5) and STZ group (n=10). STZ group mice were treated with STZ in the 1^st^ week at the dose of 50 mg/kg i.p. for 3 consecutive days. After 15 weeks all animals were sacrificed and a pair of kidneys from each mouse were dissected out and preserved for further studies. In experiment #4, we used the heterozygous and homozygous Gα_12_-deficient mice and Gα_13_LKO mice as generated in the previous experiments 7, 11 which were subjected to STZ injections as above (a 13 weeks model) and the kidney samples were analyzed in a similar way.

### Biochemical analysis

After 18 weeks, overnight fasted mice were euthanized and blood was collected into non-EDTA tubes. Serum was separated by centrifugation (4000 rpm, 25 °C, 15 min) and stored at -80 °C. Blood urea nitrogen (BUN), creatinine (Cre) and blood glucose levels were measured using a FUJI DRI-CHEM 7000 (Fujifilm, Tokyo, Japan).

### Immunoblot analysis

Immunoblot analysis of the mouse kidney tissue was done as per established laboratory protocols. Samples were selected randomly on the basis of blood glucose levels. In brief, the cortex and medulla were separated from the kidney, and protein lysates were prepared using RIPA lysis buffer. Later, protein samples were subjected to SDS-PAGE at varying percentages (7.5%, and 12%) under constant voltage. For protein transfer, ampere was kept constant, and nitrocellulose membranes (AmershamTM, ProtranTM, 10600001, Germany) were used with standard transfer buffer. To prevent non-specific binding, membranes were blocked with 5% skim milk (DifcoTM, 106861, France) in tris-buffered saline with Tween 20 (TBST) for 1 h. Thereafter, membranes were rinsed with TBST. Specific primary antibodies were applied to the respective membranes and incubated overnight at 4 °C.

After the incubation, membranes were washed with TBST. Subsequently, a horseradish peroxidase-conjugated anti-mouse IgG secondary antibody was applied at room temperature with agitation. Membranes were washed again with TBST and the bands were visualized. β-actin was utilized as an internal control for densitometric normalization.

### Histopathology analysis

To prepare kidney samples for histological analysis, mice kidneys were washed with ice-cold PBS and then immersed in 4% paraformaldehyde for 24 h. Processed tissues were embedded in paraffin to enable sectioning. Tissue morphology was evaluated through either Periodic Acid-Schiff staining (PAS) staining.

### Immunohistochemistry

Kidney samples, fixed in 10% formalin, were cut into sections followed by placing them on slides. To assess the expression of α-SMA, fibronectin, and vimentin, immunohistochemical staining was performed on the kidney tissues. Briefly, paraffin-embedded sections underwent a deparaffinization process before being incubated sequentially with the appropriate primary and secondary antibodies. Following the labeling procedure, the slides were visualized and captured using microscopy.

### Electron microscopy

Kidney specimens were fixed for 1 h in a primary fixative solution containing 2.5% glutaraldehyde and 2% paraformaldehyde in 0.1 M sodium cacodylate buffer (pH 7.2), followed by overnight incubation at 4°C. Secondary fixation was performed using 1% osmium tetroxide and 1.5% potassium ferricyanide in the same buffer, and tissues were dehydrated through a graded ethanol series. The dehydrated tissues were infiltrated with a mixture of Epon resin and propylene oxide, embedded in Epon resin, and polymerized. Ultrathin sections (70-90 nm) were cut using a Leica UCS ultramicrotome equipped with a diamond knife and collected on copper grids. Images were acquired at a magnification of ×6000 using a transmission electron microscope at the National Instrumentation Center for Environmental Management (NICEM), Seoul National University, Seoul, Republic of Korea.

### Cell culture

HEK293 and HK-2 cells purchased from the ATCC, and HepG2 cells procured by Korean Cell Line Bank were cultured in either low-glucose or high-glucose DMEM supplemented with 10% fetal bovine serum (FBS) (Gibco, USA) and 1% penicillin-streptomycin solution (Cytiva, USA). The cells were maintained in an incubator at 37 °C with 5% CO_2_ and 95% O_2_. Cells were seeded in 6-well plates at a density of 2 × 10⁵ cells/well, and wells reaching 70-80% confluence were used. Human CiGEnC cells, a conditionally immortalized glomerular endothelial cell line, were cultured at the permissive temperature of 33°C in EGM-2 endothelial basal medium (CC-3162; Lonza) supplemented with EGM-2 SingleQuots supplements, 10% FBS and 1% penicillin-streptomycin solution. Cells were seeded into 6-well plates and, upon reaching approximately 40% confluence, were shifted to the non-permissive temperature of 37°C for 24 h to induce growth arrest.

The treatment groups are: 1) low glucose media (LG, 5 mM); 2) LG + palmitic acid (PA) 250 μM; 3) high glucose (HG, 30 mM); and 4) HG + PA 250 μM for the indicated times. For the gain-of-function studies, either CiGEnC or HEK293 cells were transfected with a Gα_12_-encoding plasmid (0.5 μg/mL) for 24 h using Lipofectamine^TM^ 3000 Transfection Reagent according to the manufacturer's instructions and then treated with HG+PA for 6 h or 1.5 h after 3 h recovery period, respectively. For the loss-of-function studies, cells were transfected with 0.5 μg/mL Gα_12_ siRNA or control siRNA (siCON) for 48 h to knockdown the protein. These cells were similarly treated with HG+PA, and used for immunodetection assays.

For immunoblotting, the cells were lysed by adding lysis buffer, and the resulting lysates were incubated on ice for 1 h. After incubation, the lysates were centrifuged at 10,000*g* for 10 min, and the supernatants were collected. Proteins were separated by 7.5% or 12% SDS-PAGE and transferred to nitrocellulose membranes. Membranes were blocked with 5% non-fat dried milk in Tris-buffered saline with 0.1% Tween 20 (TBST) for 1 h at room temperature, followed by overnight incubation at 4 °C with the primary antibody. After washing with TBST buffer, the membranes were incubated with HRP-conjugated anti-mouse IgG secondary antibody for 1 h at room temperature. Protein bands were visualized. Equal protein loading was confirmed by immunoblotting for β-actin. Quantification was performed by scanning densitometry and normalized to β-actin levels.

### Immunofluorescence

For immunofluorescence, HEK293 cells were seeded using poly L-lysine-coated slides followed by fixing with 4% (w/v) paraformaldehyde (PFA) for 10 min at room temperature. After fixation, the cells were washed three times with PBST (0.1% Tween-20). Later, cells were permeabilized, and 1% BSA/ 10% normal goat serum/0.3 M glycine in PBST was added for blocking for 1 h at room temperature. The cells were then washed with PBST, and slides were incubated with primary antibody for fibronectin overnight at 4 °C. Afterwards the cells were washed and incubated with secondary antibody (Alexa-488-conjugated anti-Rabbit) for 1 h at room temperature. Subsequently, mounting medium VECTASHIELD® containing DAPI was added for slide mounting. Images were visualized using confocal microscope.

### RNA-seq analysis

For RNA-seq analysis, kidneys were collected from wild-type (WT) mice fed either a ND with vehicle injections or a HFD with STZ injections on 12^th^ week (3 injections) of total duration of 18 weeks (n = 5 for ND; n = 4 for HFD+STZ). Three QC-passed samples from each group were selected for RNA-seq analysis 7. Publicly accessible patients' expression data, downloaded from Gene Expression Omnibus (GEO, https://www.ncbi.nlm.nih.gov/geo/; GSE30528), were additionally used. Differentially expressed genes (DEGs) were identified using an independent t-test: DEGs were selected as the genes with *P*-values < 0.05 with absolute fold-change of > 1.5. The criterion for statistical significance was set at FDR < 0.25. Statistically enriched signaling pathways of clustered DEGs were ranked and categorized according to the 'Gene ontology pathway', Reactome pathway', and 'WikiPathway' using DAVID software, and DAVID Knowledgebase v2024q4 (https://davidbioinformatics.nih.gov/). Each gene represented by an individual dot in a volcano plot and a heat map of significantly expressed genes were obtained by GraphPad Prism 9.5.0 and R software/Bioconductor package using ggplot and gplots function for the visualization.

### Gene set enrichment analysis

Transcriptome data from mice fed on HFD with STZ for 18 weeks were analyzed using Gene Set Enrichment Analysis (GSEA) 4.3.3 software. 'Hallmark', 'WikiPathways”, 'Reactome pathways', and 'Biocarta pathway' from Molecular Signature Database (MSigDB v2025.1.Mm, http://software.broadinstitute.org/gsea/msigdb) and GSEA leading-edge analysis were employed using GSEA 4.3.3 software with the “Signal2Noise” metric to generate a ranked list and a “gene set” permutation type. FDR was used for the statistical significance assessment of the normalized enrichment score (NES). Gene sets with FDR < 0.25 were considered statistically significant. Heatmap represents the respective leading-edge subsets of the most upregulated genes.

### Statistical analysis

Statistical significance was tested via two-tailed Student's t-test, one-way ANOVA. For post hoc multiple comparisons following ANOVA, either the least significant difference (LSD) test or Tukey's multiple comparison test. For microscopic analysis of the cells two-way ANOVA coupled with Dunnett's test was applied. Statistical associations were evaluated using Pearson's correlation coefficients (r). For all analyses, significance was defined at thresholds of p < 0.05 (*), p < 0.01 (**), and p < 0.001 (***). Data processing and statistical computations were conducted using IBM SPSS (version 21) and GraphPad Prism (version 8.0) software.

## Results

### ER stress induction in the kidney by HFD+STZ and correlation with Gα_12_ Expression

**Figure 1:**
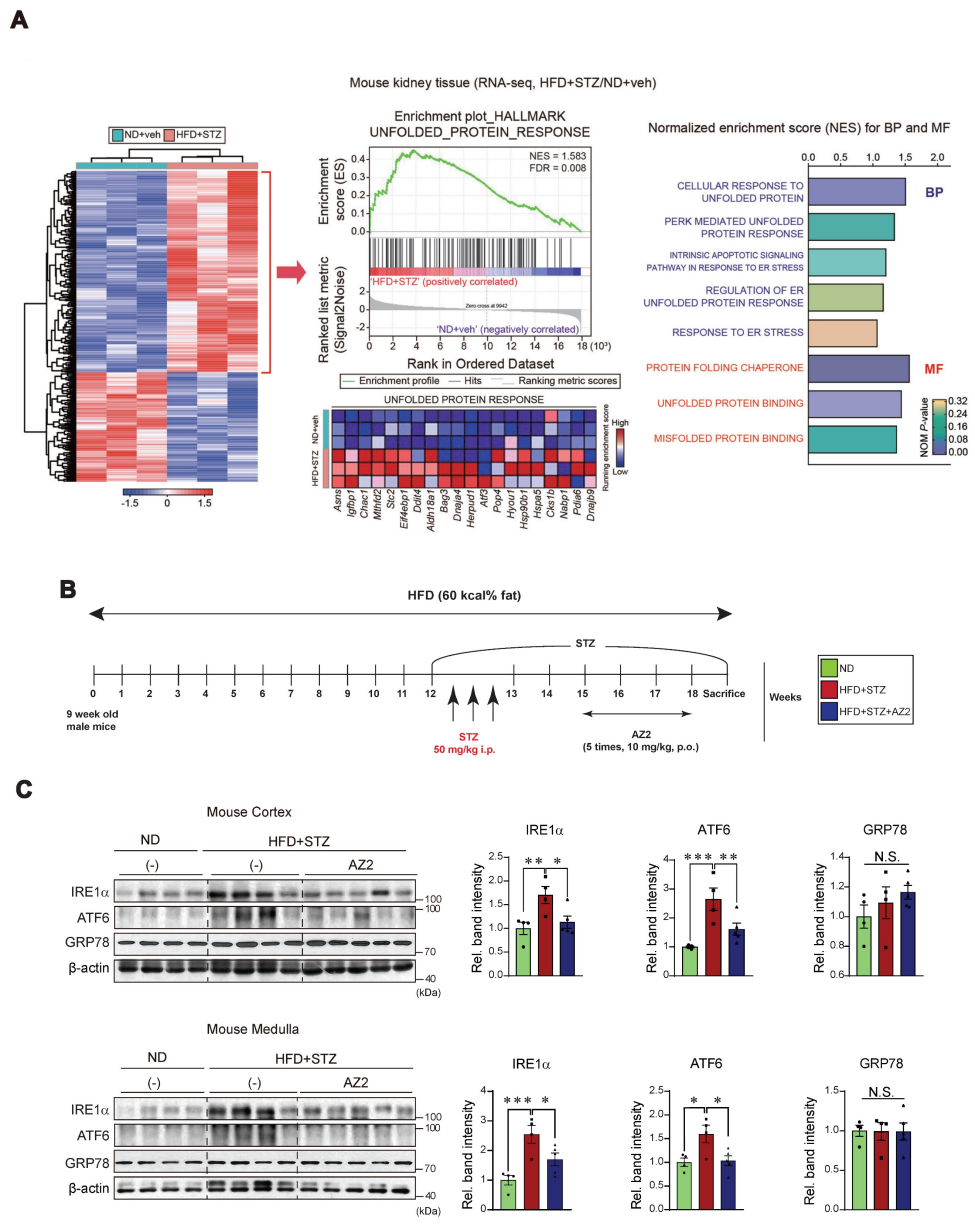
** Induction of ER stress in the kidney (A)** Heatmap hierarchical correlation analysis of differentially expressed genes (DEGs) (absolute fold-change >1.5 and *P* <0.05) (left) and GSEA-enrichment plot of the Hallmark category showing the unfolded protein response (UPR) (NES = 1.583, FDR = 0.008) positively correlated with HFD+STZ treatment in kidney tissue using transcriptome data (n = 3 each) (middle). The top 20 genes comprising the enrichment score's leading edge are indicated in the corresponding heatmap (blue, low; red, high). Bar graphs (right) of significantly enriched UPR-related signaling pathways positively correlated with HFD+STZ-treated WT mice in GSEA biological process and molecular function. NES and NOM *P*-value are shown in the bar graph. **(B)** Schematic of the induction and treatment protocol. Diabetes was established by feeding mice a 60% HFD for 18 weeks, with the addition of STZ injections (50 mg/kg body weight, i.p three times) at the 12th week. The AZ2 compound (10 mg/kg body weight) was administered orally five times weekly starting at week 15 and continuing until week 18. **(C)** Immunodetection assays for IRE1α, ATF6, and GRP78 were done using kidney cortex (upper) and medulla (lower) homogenate samples. For** C**, β-actin was used as normal control for densitometric analysis. The quantification of relative band density is provided for the indicated samples (n = 4 or 5 per group). Results represent the mean ± SEM. One-way ANOVA combined with LSD *post-hoc* analysis was utilized to calculate significance (*p < 0.05, **p < 0.01, ***p < 0.001). Groups with no significant difference are labeled N.S. (not significant).

Previously, we found that HFD feeding caused alterations in the levels of FFAs not only in the skeletal muscles and adipose tissue, but also in the blood, which was entirely reversed by genetic deletion of USP21 via AMPK 12. To identify key regulators involved in hyperglycemia-induced kidney injury, we first performed RNA-seq analysis using kidney tissues from mice treated with HFD+STZ and found that HFD+STZ treatment induced a marked shift in the global transcriptional profile, as illustrated in the heatmap (Figure 1A, left). Notably, a number of differentially expressed genes (DEGs) were associated with UPR, which ranked among the top 20 most enriched gene sets in HFD+STZ-treated mice compared to control (Figure 1A, middle). In particular, gene sets related to UPR pathways were significantly enriched in HFD + STZ-treated kidneys (Figure 1A, right).

**Figure 2:**
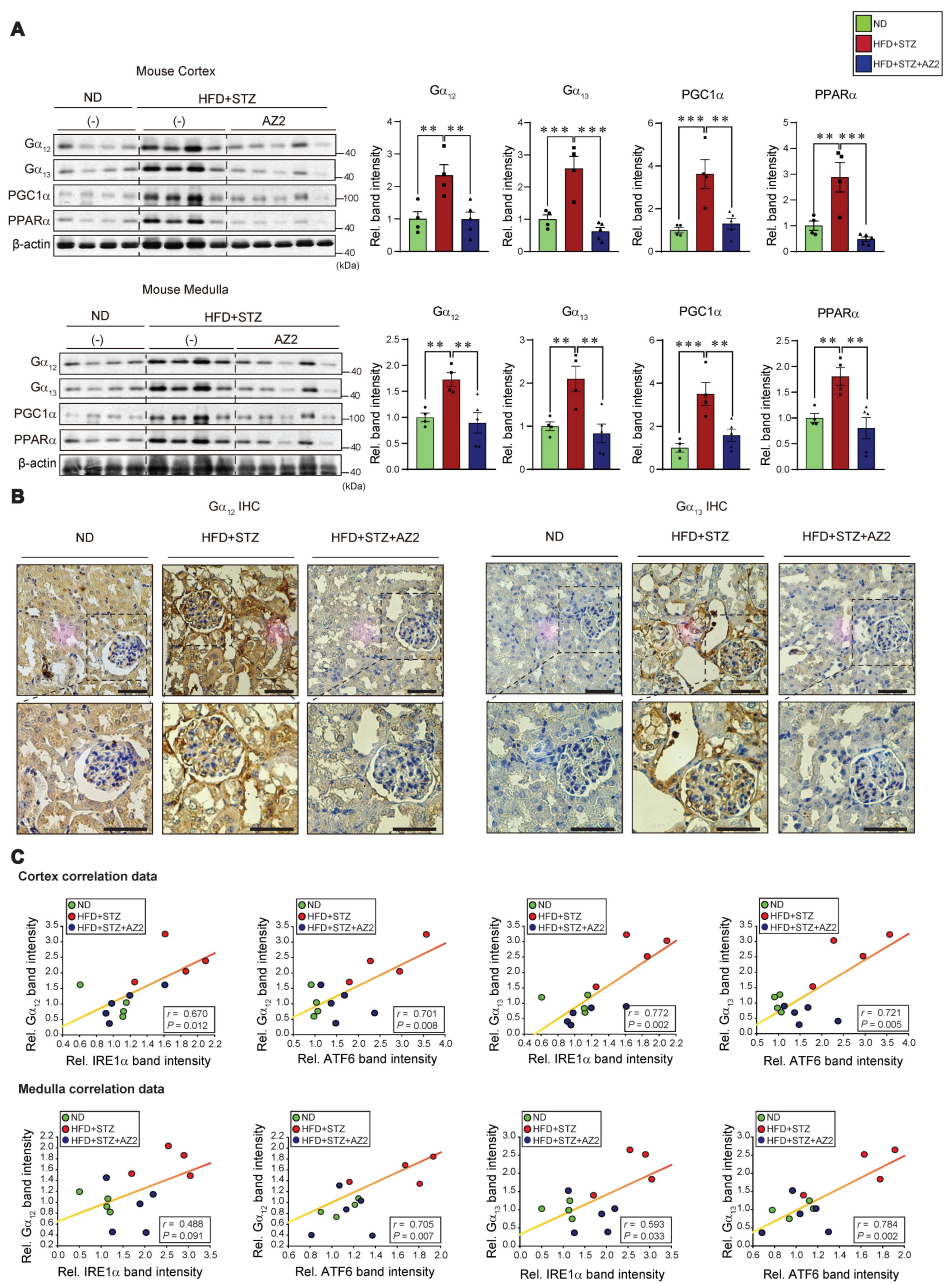
** Gα_12_ family overexpression in the kidney. (A)** Immunodetection of Gα_12_, Gα_13_, PGC1α, and PPARα was carried out on the homogenates used in Figure 1, with band intensity quantification provided for n= 4 or 5 per group. **(B)** Representative IHC images showing Gα_12_ and Gα_13_ localization; scale bar represents 100 μm. (n= 4 or 5 per group)** (C)** Pearsons's correlation were calculated to determine the correlation between Gα_12_ or Gα_13_ and either IRE1α or ATF6 (n= 4 or 5/group). For **A**, β-actin was used as normal control for densitometric analysis. Values are represented as mean ± SEM (**p < 0.01, ***p < 0.001, and N.S., not significant). One-way ANOVA in association with an LSD multiple comparison was used for calculating statistical significance (**A**).

Given the changes in UPR-associated genes, we next examined the effect of HFD+STZ on ER stress markers in the kidney cortex and medulla (Figure 1B), and observed that the levels of IRE1 and ATF6 were both increased, but not GRP78 (Figure 1C). This ER stress response was prevented by AZ2 treatments. Previously, we showed that severe ER stress is accompanied by Gα_12_ overexpression in the livers of different animal models (i.e., APAP intoxication or HFD+STZ) 7, 13. So, we examined the expression of Gα_12_ family proteins, and found that the levels of Gα_12_ and Gα_13_ were upregulated in cortex and medulla (Figure 2A). Parallel to these changes, we confirmed increases of PGC1α and PPARα in the samples, indicative of the adaptive changes in mitochondrial fuel consumption. AZ2 treatments restored the levels of Gα_12_, PGC1α, and PPARα to normal. Immunohistochemical analyses validated the HFD+STZ-induced increases of Gα_12_ and Gα_13_ (Figure 2B). In addition, correlations between IRE1 or ATF6 and Gα_12_ family proteins were demonstrated in both kidney cortex and medulla (Figure 2C). These results show that HFD+STZ treatments resulted in sustained ER stress accompanying Gα_12_ overexpression in kidney tissue.

### NLRP3 inflammasome activation by HFD+STZ and correlation with Gα_12_ overexpression

**Figure 3:**
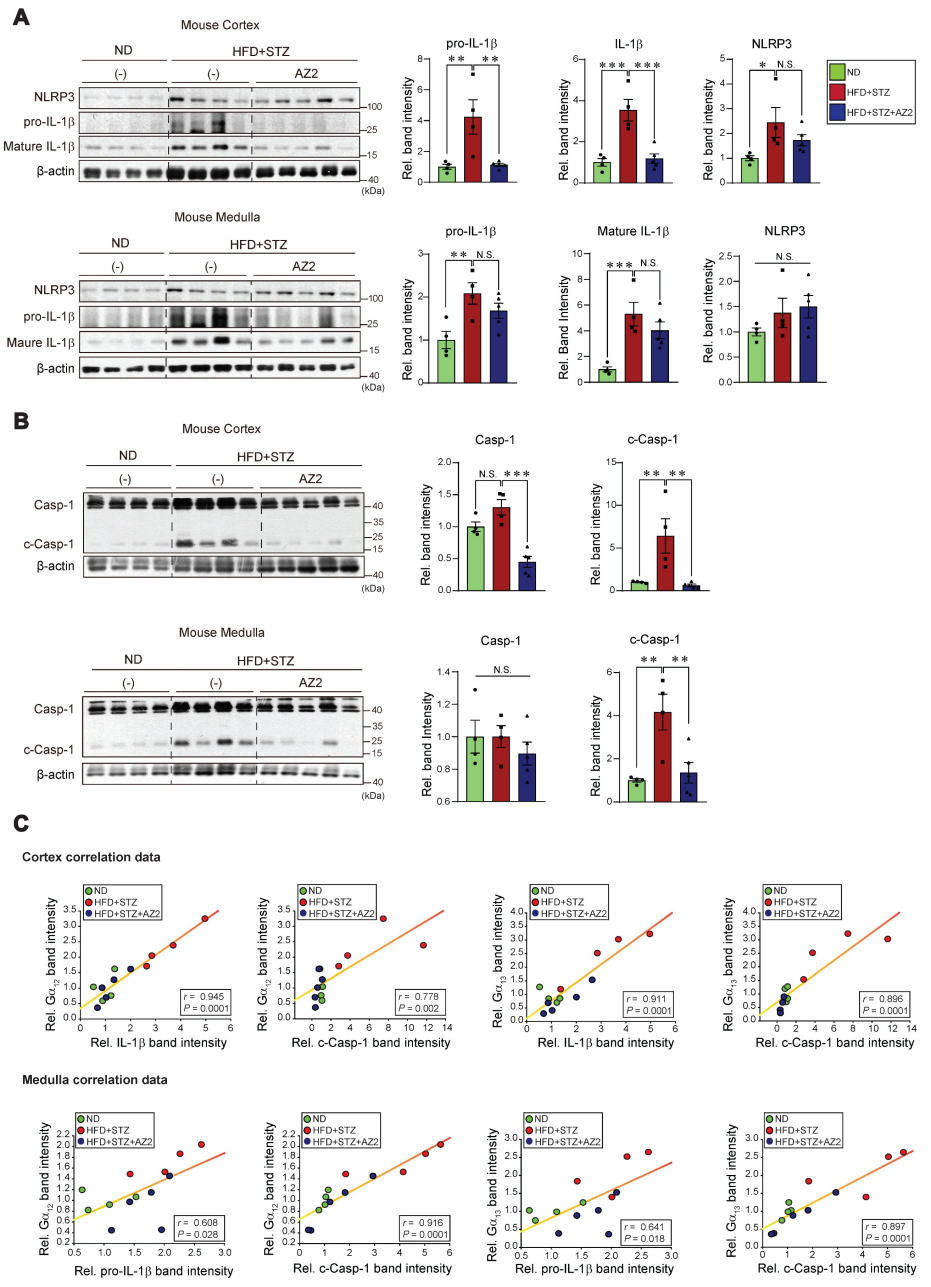
** Pyroptosis induction in the kidney. (A)** Immunodetection assays for NLRP3, pro-IL-1β and IL-1β were performed using the same kidney samples. **(B)** Immunodetection assays for Caspase-1 (Casp-1) and cleaved Caspase-1 (c-Casp-1) were done. (**C**) Pearsons's correlation assays between Gα_12_, and IL-1β, pro-IL-1β, or c-Casp-1; and Gα_13_, and IL-1β, pro-IL-1β, or c-Casp-1 were performed as in Fig. **2C** (n = 4 or 5/group). For **A** and** B**, β-actin was used as normal control for densitometric analysis. The quantification of protein expression levels (n = 4 or 5 per group) is provided as the mean ± standard error of the mean (SEM). A one-way ANOVA in conjunction with an LSD *post-hoc* analysis was used to calculate significant differences. P-values are represented as follows: *p < 0.05, **p < 0.01, ***p < 0.001. N.S. indicates no statistical significance **(A, B)**.

Our previous research demonstrated an association between Gα_12_ overexpression and pyroptosis in colonic epithelial cells in an inflammatory bowel disease model 9, and hepatocyte injuries elicited by HFD+STZ treatments 13. We next examined key markers of pyroptosis in the kidney. Notably, sustained ER stress caused by HFD+STZ increased the levels of NLRP3, pro-IL-1β and mature IL-1β (Figure 3A). The increases of pro-IL-1β and mature IL-1β were reversed by AZ2 treatments although the increase of NLRP3 was not significantly decreased. Consistently, we observed increases in both caspase-1 and cleaved caspase-1 levels (Figure 3B). Particularly, cleaved caspase-1 levels were highly elevated by HFD+STZ treatments. So, the ability of AZ2 to inhibit IL-1β and caspase-1 activation may rely on the step downstream of NLRP3 (i.e., inflammasome complex). Our subsequent analyses displayed strong positive correlations between Gα_12_ (or Gα_13_) and either IL-1β or cleaved caspase-1 levels (Figure 3C), suggestive of the functional role of Gα_12_ signaling axis in NLRP3-dependent pyroptosis. Similar results were obtained when we used kidney medulla. These results provide strong evidence that HFD+STZ treatments may cause NLRP3 inflammasome activation, leading to pyroptosis of kidney tissue in association with ER stress accompanying Gα_12_ overexpression.

### Functional gene set analysis for RNA-seq dataset of the kidney

**Figure 4:**
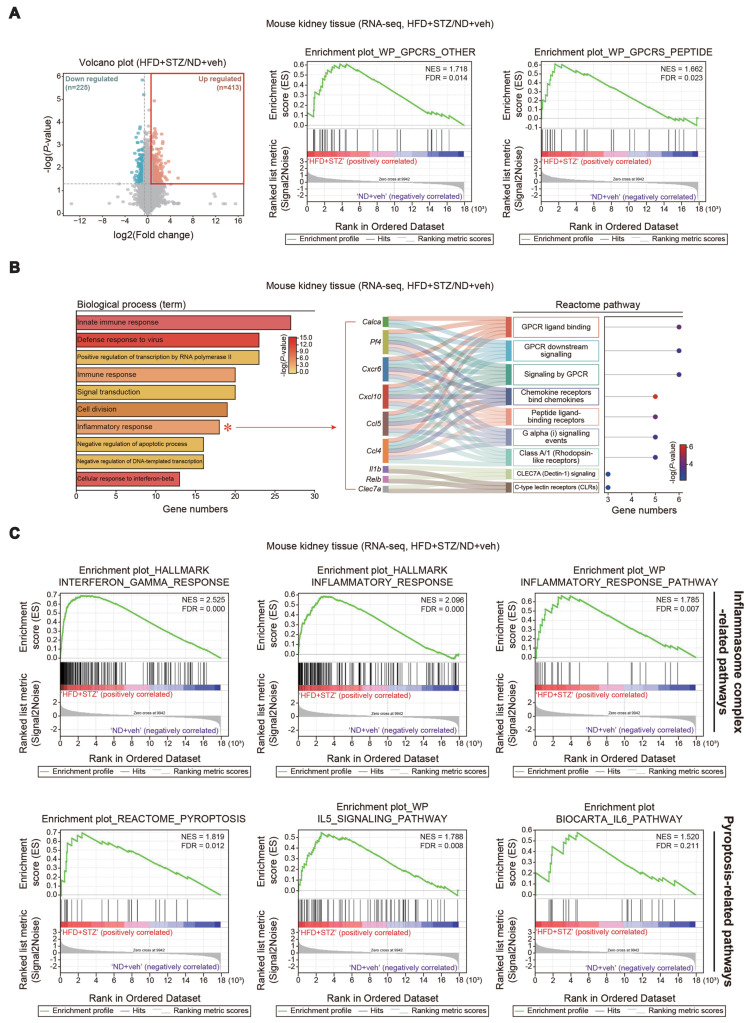
** Analyses of RNA sequence datasets from the kidney of HFD+STZ-treated mice. (A)** Volcano plot of DEGs using the same data as in Fig. **1A**. Horizontal and vertical lines indicate the filtering criteria (absolute fold-change >1.5 and *P* <0.05, respectively). Red and mint dots show upregulated (413 genes) or downregulated (225 genes) DEGs in response to HFD+STZ (left, n = 3 each). GSEA-enrichment plot of the GPCR signaling-related pathways (WikiPathways, WP) from the same data as in Fig. **1A.** (NES = 1.718, FDR = 0.014; NES = 1.662, FDR = 0.023) (right). **(B)** Bar graphs based on Gene Ontology (GO) analysis to identify biological processes regulated by HFD+STZ using the same data as in Fig. **1A**. Inflammatory response (red asterisk) showed a great number of associated genes (left). (DEGs of *P*-value <0.05 and FC >1.5). *P*-value and gene numbers are presented as bar graphs. GPCR signaling-related pathways were identified based on the Sankey diagram (plot) generated from the leading 9 genes associated with the inflammatory response in the biological process category. The Sankey diagram represents genes within each pathway; dot colors display *P*-values. **(C)** GSEA-enrichment plot of the inflammasome complex-related pathways (Hallmark and WikiPathways, WP) and pyroptosis-related pathways (REACTOME, WP, and BIOCARTA) from the same data as in Fig. **1A**.

To assess ER stress and cell signaling pathways in the context of transcript levels, we further analyzed RNA-seq data from kidneys of mice treated with HFD+STZ. DEGs accounted for approximately 3.4% of the entire transcriptome; among the 638 DEGs identified, 225 genes were downregulated and 413 were upregulated (Figure 4A, left). Gene set enrichment analysis (GSEA) of the kidney transcriptome revealed a significant enrichment of gene sets associated with G protein-coupled receptor (GPCR) signaling-related processes in HFD+STZ-treated mice (Figure 4A, right). Further Gene Ontology (GO) analysis of the same dataset showed that the inflammatory response pathway contained a significantly higher number of genes in the HFD+STZ group compared to control (Figure 4B). Specifically, the inflammatory response pathway comprised 18 genes predominantly associated with GPCR signaling pathways, as visualized by Sankey diagram (Figure 4B), suggesting a potential link between GPCR and ER stress in hyperglycemia-induced kidney injury. Furthermore, GSEA revealed significant enrichment of inflammasome complex-related gene sets within the HALLMARK and WikiPathways, as well as pyroptosis-related gene sets from the REACTOME, WikiPathways, and BIOCARTA (Figure 4C). These results together with our experimental outcomes support the functional role of Gα_12_ overexpression and ER stress in the inflammasome pathways and the accompanying pyroptosis in the kidney of HFD+STZ-treated mice.

### Glomeruli and podocytes changes, and renal dysfunction by HFD+STZ

**Figure 5:**
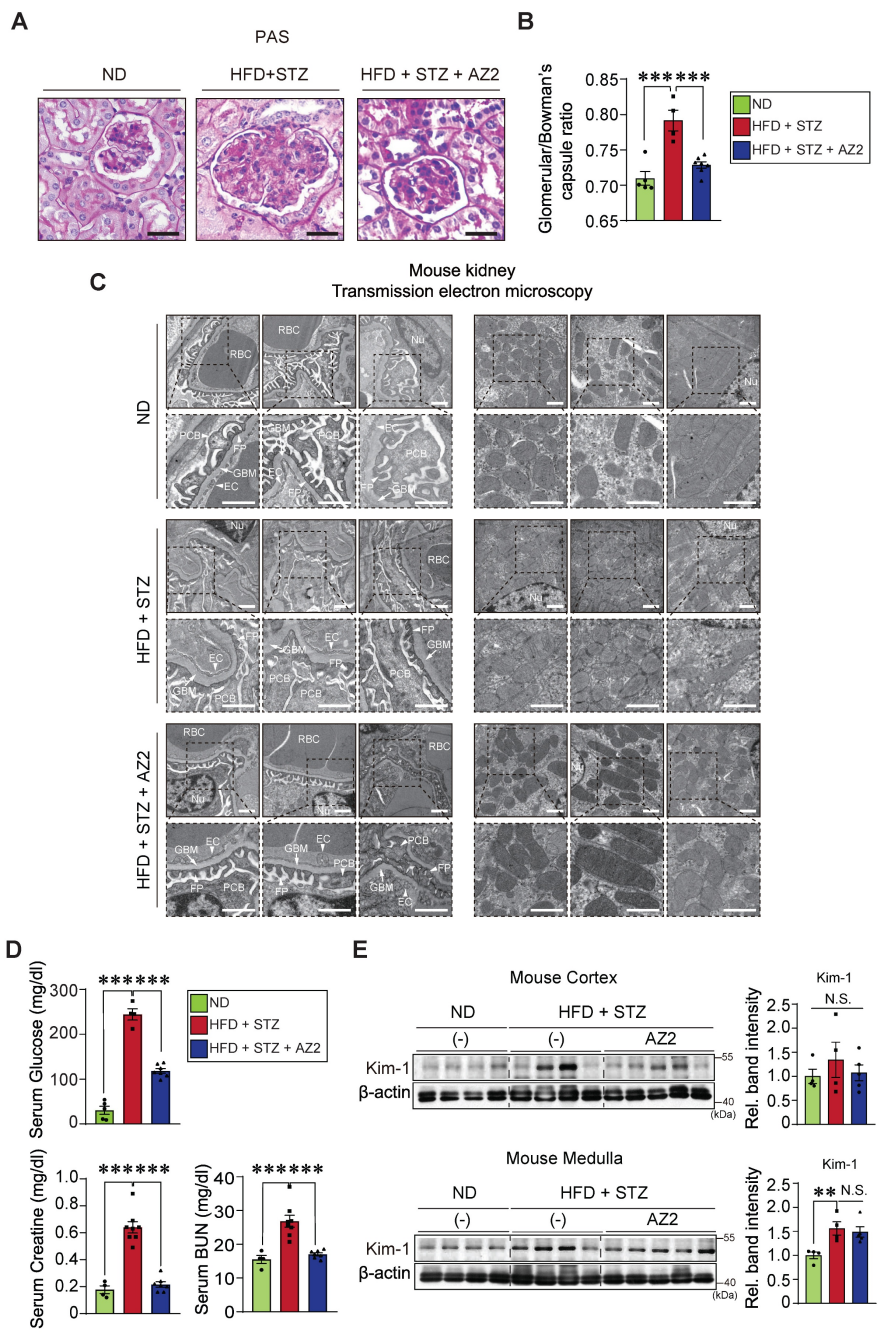
**Histochemical assays and transmission electron micrographs, and kidney function and injury marker levels.** (**A**) PAS staining of kidney morphologies of the samples as in Fig. **1** (n = 5, 4 or 7/group). Scale bar: 100 μm. (**B**) The bar graph shows the glomerular/Bowman's capsule ratio of the kidney (n = 5, 4 or 7/group). Each point represents the mean value of 15 arbitrary visual fields in each kidney sample. (**C**) Representative TEM of the kidney (n = 5, 4 or 7/group) (Scale bar, 1 µm; EC, endothelial cell; FP, foot process; GBM, glomerular basement membrane; Nu, nucleus; PCB, podocyte cell body; RBC, red blood cell). **(D)** Random blood sugar (RBS; serum glucose) (n = 5, 4 or 7/group), CRE, and BUN contents in the mice (n = 4, 9 or 7/group). **(E)** Immunodetection assays for Kim-1 for kidney cortex (upper) and medulla (lower) (n = 4 or 5/group). For comparison of band intensities within the blots, 4 or 5 samples were chosen based on blood sugar content. For **E**, β-actin was used as normal control for densitometric analysis. The quantification of band intensities is given to their corresponding samples (n = 4 or 5/group). Values are represented as mean ± SEM (**p < 0.01, ***p < 0.001, and N.S., not significant). One-way ANOVA in association with an LSD multiple comparison was used for calculating statistical significance (**B, D, E**).

Having identified pyroptotic cell death in the kidney by chronic treatments with HFD+STZ, we were next interested in whether hyperglycemia and fat overload induce glomeruli and podocytes alterations morphologically, and if so, what renal functions would be. As expected, histopathological analyses revealed enlargement of glomeruli, accumulation of large vacuoles, and tubular degeneration in mice subjected to HFD+STZ treatments (Figure 5A). Multiple analyses of renal glomeruli, quantified by assessing 15 glomeruli per sample, verified significant increases of glomeruli/Bowman's capsule ratios (i.e., from 0.71 to 0.79; 12.9% change) (Figure 5B). These alterations were diminished by AZ2 treatments.

As a continuing effort to examine glomerular morphology, we employed electron microscopic approaches. TEM analyses of kidney tissues from HFD+STZ-treated mice suggested ultrastructural changes compatible with the glomerular alterations observed by light microscopy (Figure 5C, left). The images often showed accumulation of relatively large vacuoles and regions with broadened or less clearly demarcated podocyte foot processes, which may reflect structural impairment of the glomerular filtration barrier. Furthermore, TEM analysis revealed mitochondrial swelling and fragmentation with disrupted cristae in kidneys from HFD+STZ-treated mice, which may indicate mitochondrial dysfunction (Figure 5C, right). In the AZ2-treated group, these mitochondrial and podocyte alterations appeared less prominent, suggesting a potential attenuation of ultrastructural injury. Overall, these findings are compatible with HFD+STZ-induced kidney damage and raise the possibility that AZ2 treatment exerts a protective effect.

Given the increases in pyroptotic cell injury and morphological alterations in glomeruli/Bowman's capsule, we then examined blood biochemical parameters of kidney function and molecular biomarkers. We confirmed the serum glucose contents were highly elevated in the HFD+STZ animal model (244.3 ± 12.7 mg/dl), which was accompanied by increases in the CRE and BUN contents. As expected, AZ2 treatments showed a hypoglycemic effect with CRE and BUN normalization (Figure 5D). To verify renal complications, we monitored the levels of Kim-1, and found a significant moderate increase in Kim-1 levels in the medulla (Figure 5E). Changes in the cortex were not statistically significant. AZ2 treatment had no significant effect on Kim-1 levels in the medulla. Overall, our results showed that HFD+STZ treatments elicited alterations in glomeruli and podocyte changes, causing renal dysfunction, which can be antagonized by AZ2 treatments.

### Fibrosis of kidney cortex and medulla by HFD+STZ

**Figure 6:**
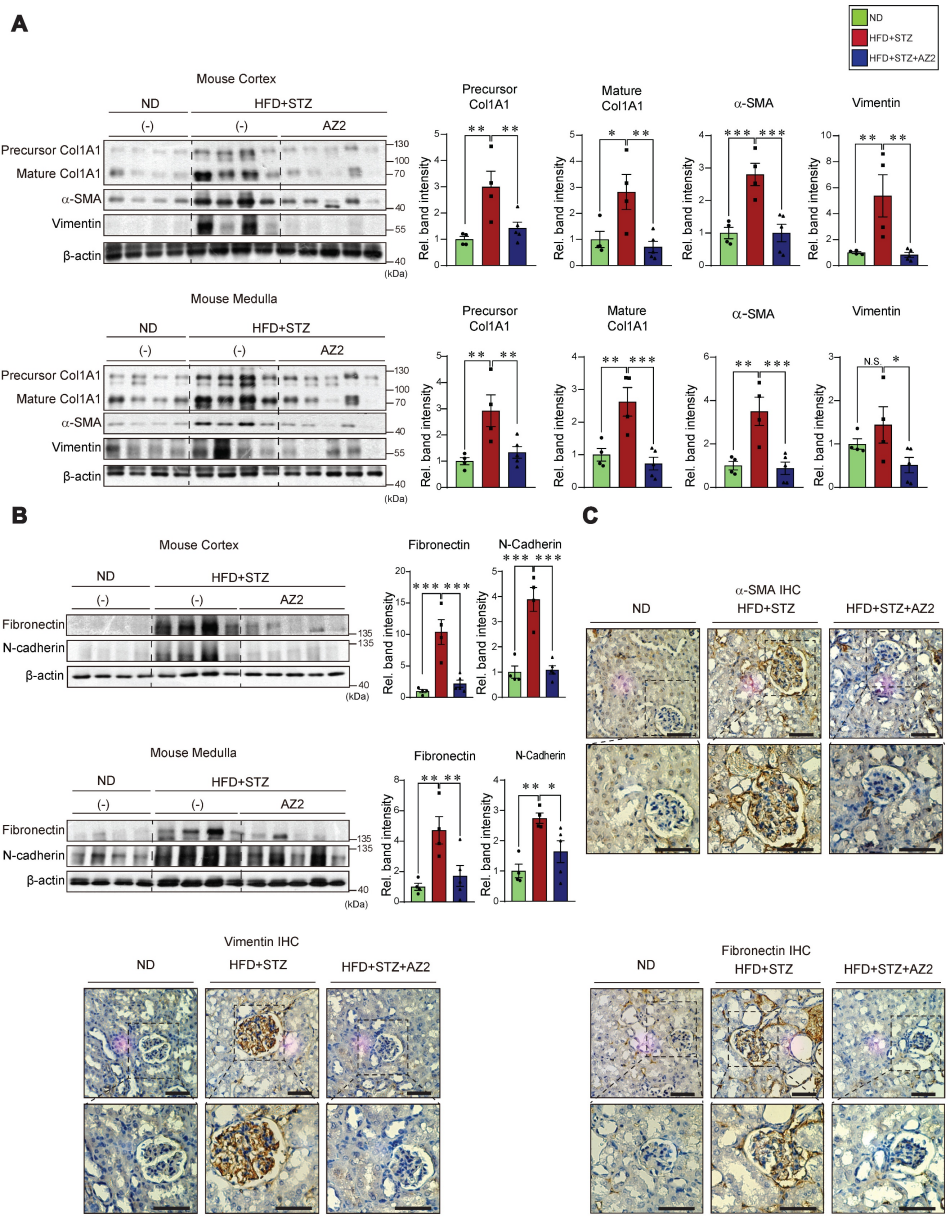
** Expression of fibrotic markers in the kidney.** (**A**) Immunodetection assays for precursor Col1A1, Col1A1, α-SMA, and vimentin were done using the same **homogenates** as in Fig. 1. (**B**) Immunodetection assays for fibronectin, and N-cadherin. (**C**) Representative IHC staining for markers of fibrosis (α-SMA, vimentin, and fibronectin) in the indicated samples (n = 4 or 5/group; scale bar: 100 μm). For the protein assays in A and B, β-actin was employed as the normalization control. Quantification graphs (n = 4 or 5/group) depict the mean ± SEM. Statistical differences were analyzed using one-way ANOVA in conjunction with an LSD multiple comparison test (*p < 0.05, **p < 0.01, ***p < 0.001). Non-significant results are marked N.S. (**A, B**).

In subsequent experiments, we assessed the effects of HFD+STZ on renal fibrosis; The levels of fibrotic markers including precursor and mature collagen 1A1, α-SMA, and vimentin were all elevated in both cortex and medulla (Figure 6A). Fibronectin belongs to key ECM glycoproteins that play a role in tissue remodeling, cell adhesion, and fibrosis 14. Thus, excess fibronectin deposition may represent glomerular and interstitial fibrosis along with renal cell atrophy 15. In the immunoblotting assays, we observed pronounced increases of fibronectin in not only the kidney cortex, but also the medulla (Figure 6B). Consistently, the expression levels of N-cadherin were elevated. All of these changes were diminished by treatments with AZ2.

To confirm the morphological changes, we performed immunohistochemistry, and verified that α-SMA, vimentin, and fibronectin accumulation were distinct in glomerular and surrounding areas in the mice exposed to HFD+STZ, and again these effects were notably ameliorated by AZ2 (Figure 6C). Together, these results support the notion that chronic hyperglycemia in conjunction with fat overload facilitates fibrotic complications and that AZ2 is pharmacologically effective.

### Mild complications in the kidney of mice treated with STZ alone

**Figure 7:**
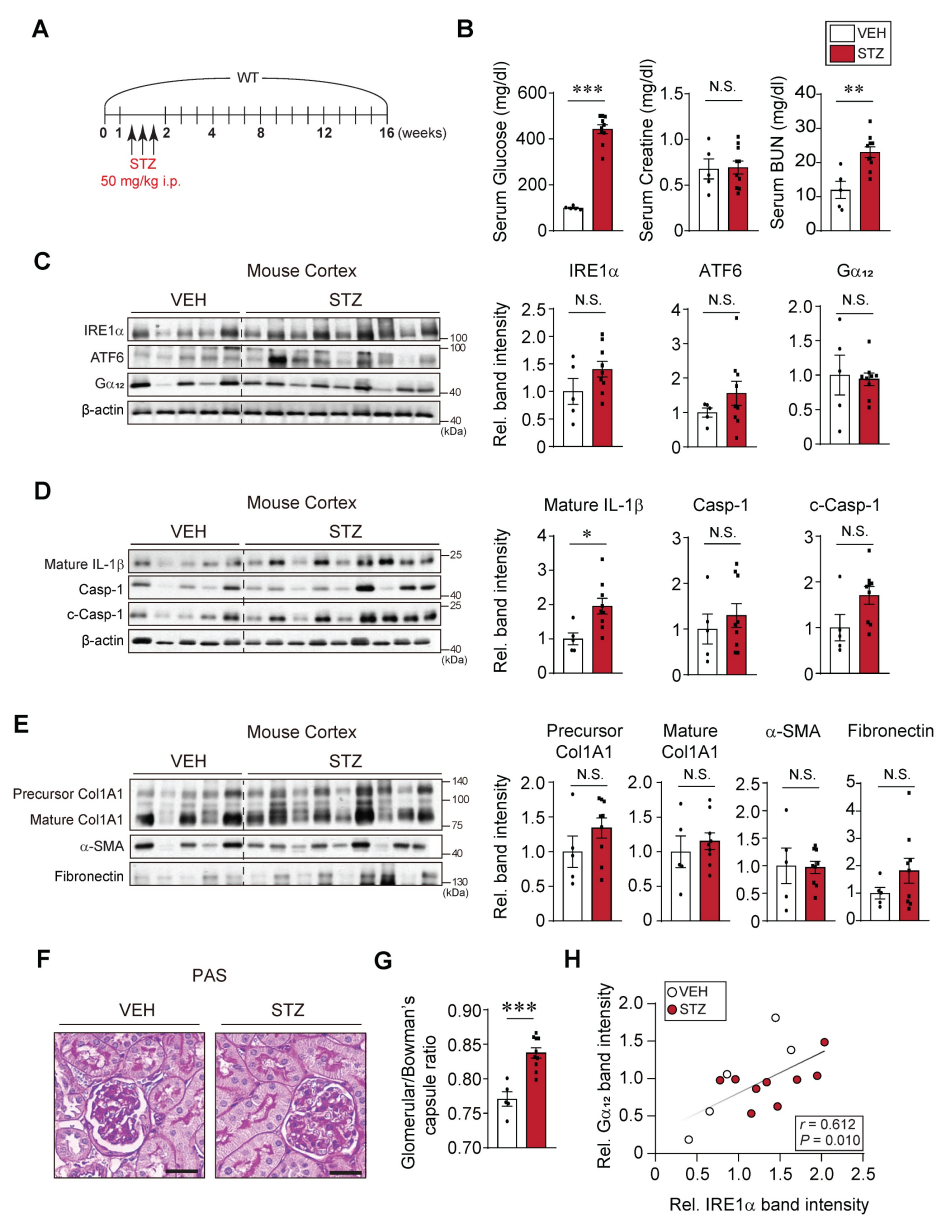
** Effects of STZ treatments on ER stress, pyroptosis and fibrosis markers in the kidney. (A)** A schematic explanation of an animal experiment showing mice subjected to STZ treatments (50 mg/kg body weight, i.p., 3 times on 1^st^ week and sacrificed after 15^th^ week). (**B**) Serum RBS, CRE, and BUN contents (n = 5 or 10/group). (**C**) Immunodetection assays for IRE1α, ATF6 and Gα_12_. (**D**) Immunodetection assays for mature IL-1β, Casp-1 and c-Casp-1. (**E**) Immunodetection assays for Col1A1, α-SMA and fibronectin of the same samples. (**F**) PAS staining of the kidney. Scale bar: 100 μm. (**G**) The bar graph shows the glomerular/bowman's capsule ratio of the kidney (n = 5 or 10/group). Each point represents the mean value of 15 arbitrary visual fields in each kidney sample. For comparison of band intensities within the blots, 5 or 9 samples were chosen for immunoblottings based on blood sugar content. (**H**) Pearsons's correlation assay between Gα_12_ and IRE-1α was performed as in Fig. **2C** (n = 5 or 9/group). For **C, D**, and **E,** β-actin was used as normal control for densitometric analysis. For **D** and **E**, β-actin is shared. The quantification of band intensities is given to their corresponding samples (n = 5 or 9/group). Values are represented as mean ± SEM (*p < 0.05, **p < 0.01, ***p < 0.001, and N.S., not significant). Two-tailed unpaired Student's *t* test was done for calculating statistical significance (**B-E, G**).

In comparison with the results obtained from mice exposed to HFD+STZ, we further assessed whether STZ-induced hyperglycemia also elicits similar extents of kidney complications (Figure 7A). At 16 weeks after STZ treatments, we confirmed a significant increase in blood glucose levels which were greater than that elicited by HFD+STZ (443 ± 19.2 vs. 244.3 ± 12.7 mg/dl, respectively) (Figure 7B). The content of BUN, but not CRE, was elevated, indicating that renal dysfunction caused by STZ treatment alone was less pronounced than that caused by HFD+STZ. We also examined the representative ER stress marker levels in the cortex, and found no significant changes (Figure 7C). Of note, the expression levels of Gα_12_ were not increased, which was in sharp contrast to that elicited by HFD+STZ treatments. In this experimental set, we mainly used the kidney cortex tissue for immunoblotting assays because there had been no significant difference in the ER stress marker changes between cortex and medulla.

When we analyzed the pyroptosis markers in the renal cortex, we observed that IL-1β levels were increased by 2-fold, which was inferior to that caused by HFD+STZ (3.5- and 5.2-fold in the cortex and medulla tissues, respectively). Moreover, pro-Casp-1 and c-Casp-1 levels were not significantly increased (Figure 7D). Consistently, there were no changes in the levels of fibrotic markers such as precursor and mature forms of Col1A1, α-SMA, and fibronectin (Figure 7E). The less pronounced kidney complication by STZ-induced hyperglycemia alone was corroborated by the assessments of histochemical glomerular morphologies (Figure 7F) and glomeruli/Bowman's capsule ratios (0.77 vs. 0.83; percent difference, 7.9%) (Figure 7G). Despite the relatively mild renal injury induced by STZ alone, a significant positive correlation was observed between Gα_12_ and IRE1α (Figure 7H). Together, these results provide strong evidence that prolonged hyperglycemia alone induces a moderate increase of IL-1β (i.e., mild pyroptosis), which may not be sufficient to cause severe renal complications such as Gα_12_ overexpression, IRE1 activation, and fibrogenesis.

### High glucose plus palmitate-induced pyroptosis in cells

**Figure 8:**
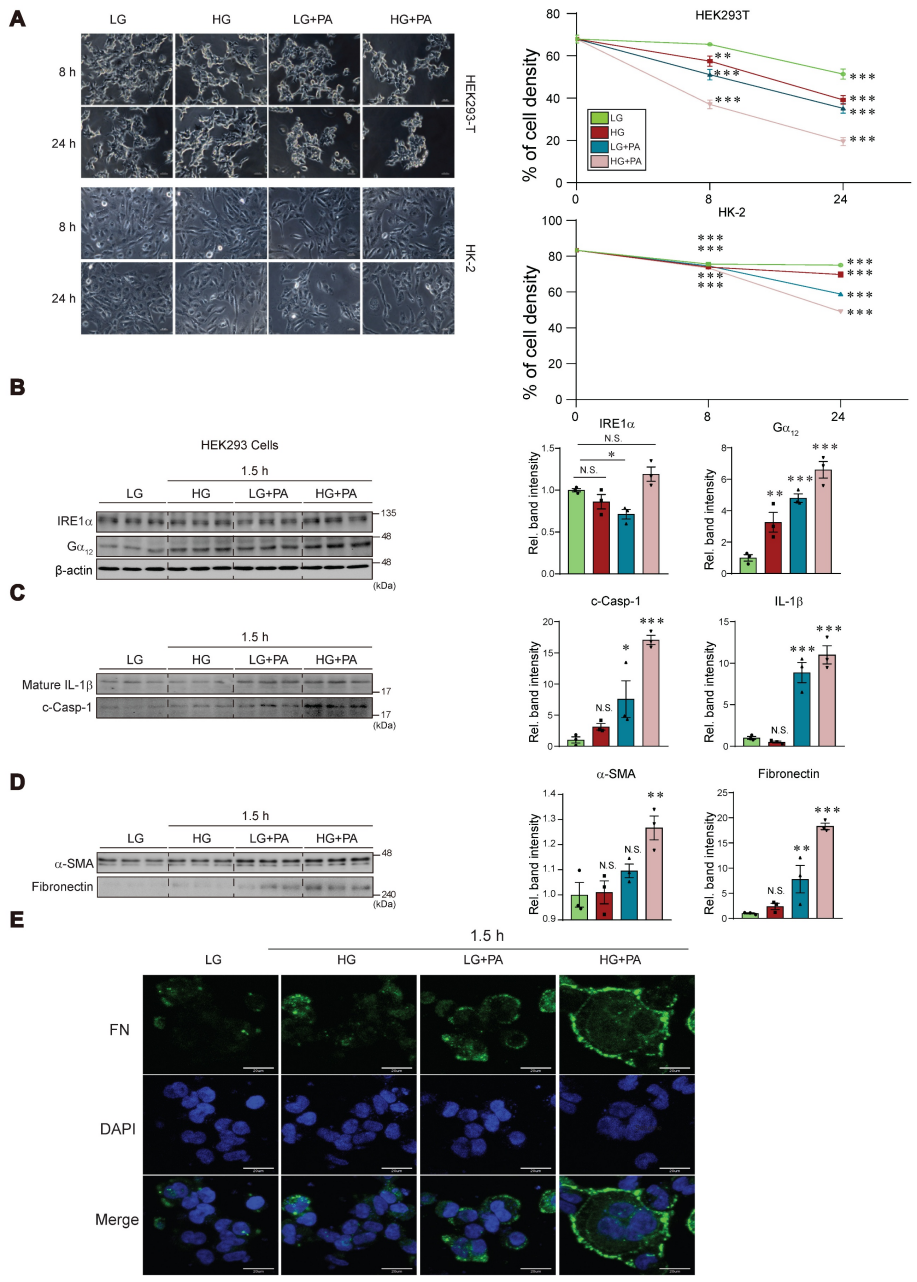
** Effects of HG and/or PA treatments on ER stress, pyroptosis, and fibrosis markers in cells. (A)** Microscopic assays of HEK293 and HK-2 cells in different time starting from 8, and 24 h (left). Quantifications of cell viability (right). The cells were treated with low glucose (LG), high glucose (HG) with or without palmitate (PA) at the indicated times. (**B**) Immunodetection assays for IRE1α and Gα_12_ in HEK293 cells (n = 3/group). (**C**) Immunodetection assays for mature IL-1β and c-Casp-1 in HEK293 cells (n = 3/group). **(D)** Immunodetection assays for α-SMA and fibronectin in HEK293 cells (n = 3/group). **(E)** Immunofluorescence assays for fibronectin in HEK-293 cells treated as above. For **B**, β-actin was used as normal control for densitometric analysis. Panels **B**, **C** and **D** share β-actin control. The quantification of band intensities is given to their corresponding samples (n = 3 each). Values represent the mean ± SEM. Statistical differences were evaluated by two-way ANOVA with Dunnett's test (panel A) or one-way ANOVA with LSD *post-hoc* analysis (panels B-D). P-values are indicated by asterisks (*p < 0.05, **p < 0.01, ***p < 0.001), and N.S. signifies a lack of statistical significance.

Previously, we have shown that Gα_12_ overexpression by toxicant-induced IRE1 activation causes ferroptosis via lipoxygenase in the liver 7. As a continuing effort to clarify the effect of high glucose (HG) and fat, we employed the *in vitro* assays using different cells. We were interested in the effects of HG plus palmitic acid (PA) on Gα_12_ expression and cell fate primarily using kidney cells. Given that incubating HEK293 kidney fibroblast (or HK-2 kidney epithelial) cells under conditions of 30 mM glucose and 250 μM PA for 8 or 24 h initiated a significant fraction of cell death (Figure 8A), we selected shorter time points of 1.5 or 3 h in subsequent experiments.

Treatment of HEK293 cells with either HG or PA alone caused weak a change in IRE1α levels as compared to that with low glucose (LG, 5 mM). Combination treatment of HG and PA tended to further increase IRE1α level (Figure 8B). Of interest, treatment with either HG or PA caused larger increases of Gα_12_, and this effect was augmented by combination treatment. Likewise, we were able to observe the largest increases in IL-1β and c-Casp-1 levels by applying HG+PA (Figure 8C). Apparently, PA effect was superior to that of HG in HEK293 cells. Consistently, the combinatorial treatment synergistically elevated α-SMA and fibronectin levels (Figure 8D). The pronounced effect of HG+PA on fibrogenesis was strengthened by immunocytochemistry of fibronectin and DAPI staining (Figure 8E).

Incubation of human kidney 2 (HK-2) cell line in 30 mM glucose with or without PA for 3 h resulted in no significant changes of IRE1α and Gα_12_ (Figure S1A). However, we could see moderate increases of IL-1β and c-Casp-1 under the conditions. When we used HepG2 cells, the combinatorial effect was clearer on all of the parameters (i.e., IRE1α, Gα_12_, IL-1β and c-Casp-1) (Figure S1B), confirming the synergistic effect of HG+PA. Collectively, these findings indicate that treating cells with HG in combination with FFA may facilitate pyroptosis and fibrosis, supporting the concept that fat overload, which leads to elevated FFAs, may exacerbate kidney complications.

### Effect of Gα_12_ modulation on high glucose plus palmitate-induced pyroptosis and fibrosis

**Figure 9:**
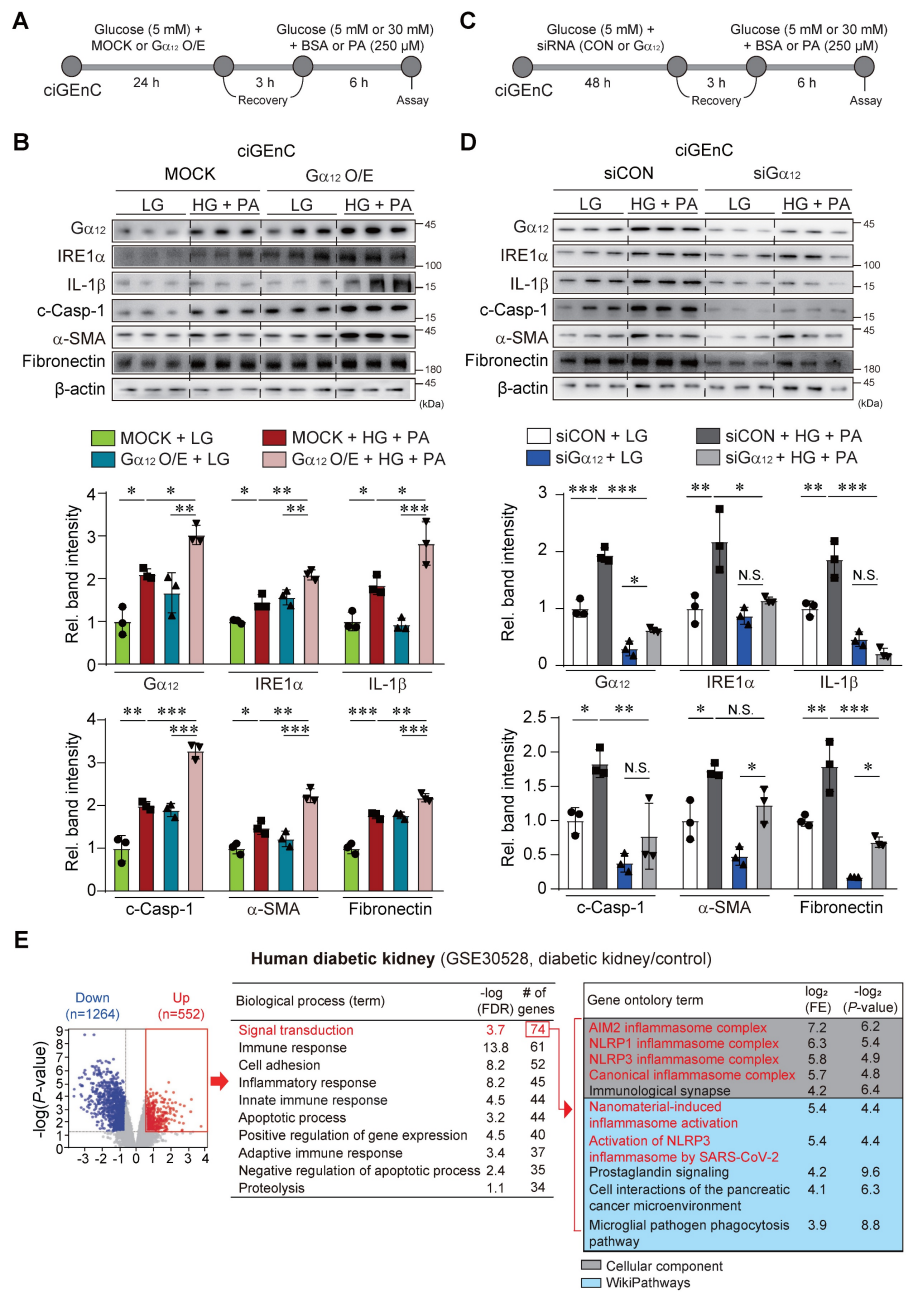
** Effect of Gα_12_ modulation on ER stress, pyroptosis, and fibrosis in HG plus PA-treated ciGEnC cells. (A)** A schematic explanation of Gα_12_ overexpression experiment. **(B)** Immunodetection assays for Gα_12_, IRE1α, IL-1β, c-Casp, fibronectin and α-SMA in the cells transfected with a Gα_12_-encoding plasmid or MOCK, and then treated with high glucose and palmitic acid (HG+PA). **(C)** A schematic explanation of Gα_12_ siRNA knockdown experiment. **(D)** Immunodetection assays in the cells treated with HG+PA after transfection with siRNA targeting Gα_12_ or siCon. **(E)** cDNA microarray (GSE30528) from patients with diabetic kidney disease (n = 9) and healthy individuals (n = 13) was analyzed. Volcano plot (left) showing DEGs (blue, downregulation; red, upregulation; DEGs with *P*-value <0.05 and absolute FC >1.5). Biological process analysis was performed using the upregulated genes identified from the volcano plot. Signal transduction pathway, ranked first, is highlighted in red (middle). GO enrichment analysis (Cellular Component and WikiPathways) based on leading genes involved in the signal transduction pathway is shown (right). Red-colored terms in the Cellular Component and WikiPathways indicate enrichment of NLRP3 inflammasome-related pathways. Values are represented as mean ± SEM (*p < 0.05, **p < 0.01, ***p < 0.001, and N.S., not significant). One-way ANOVA in association with a Tukey's multiple comparison was used for calculating statistical significance (**B, D**).

As a continuing effort to verify the effect of Gα_12_ on ER stress, pyroptosis and fibrosis, which are all processes elicited by hyperglycemia and fat overload, we treated HEK293A with HG+PA after transfection with a plasmid encoding for Gα_12_ (Figure S2A). To extend our observations to glomerular endothelial cells, we employed human CiGEnC cells that retain key phenotypic features of primary glomerular endothelial cells. Remarkably, CiGEnC cells exhibited similar results to those observed in HEK293A cells (Figure 9A). Enforced expression of Gα_12_ caused activation of IRE1α, IL-1β, c-Casp-1, fibronectin, and α-SMA in the cells incubated with LG (Figure 9B and S2B). More importantly, Gα_12_ overexpression markedly enhanced the ability of HG+PA to activate the above parameters. We then further examined the effects of Gα_12_ knockdown using siRNA (Figure 9C and S2C). As expected, the knockdown of Gα_12_ produced the opposite effects (Figure 9C, 9D, S2C, and S2D), completely preventing the increasing effects of HG+PA on ER stress, pyroptosis and fibrogenesis. These results demonstrate that the expression level of Gα_12_ directly impacts the pathological processes in response to HG and FFA.

In subsequent experiments, we analyzed a publicly available human RNA-seq dataset, and verified differentially expressed genes using a volcano plot, revealing 1264 downregulated and 552 upregulated genes in patients with diabetic kidney disease (DKD) compared to healthy individuals (Figure 9E, left). Gene ontology analysis of the upregulated gene set indicated enrichment in several biological processes, including signal transduction (the most prominently affected), immune response, cell adhesion, and inflammatory response. The signal transduction pathway comprised 74 genes, primarily associated with NLRP3 inflammasome-related pathways, as identified in Cellular Component and WikiPathways analyses (Figure 9E, right). We further carried out immunohistochemical analyses of Gα_12_ using human DKD and non-diabetic tissue samples. In DKD samples, Gα_12_ expression was increased on the margin of glomeruli presumably including the region of podocytes as compared to non-diabetic controls. The changes of IRE1 intensities were also distinct in human DKD samples (Figure S3).

All together, these results strongly support the contention that hyperglycemia in combination with FFA facilitates NLRP3 inflammasome pathways and the accompanying pyroptosis and fibrosis as mediated by Gα_12_-dependent ER stress.

### ER stress, pyroptosis and fibrosis marker levels in the kidney of Gα_12_ KO or Gα13LKO mice treated with STZ

**Figure 10:**
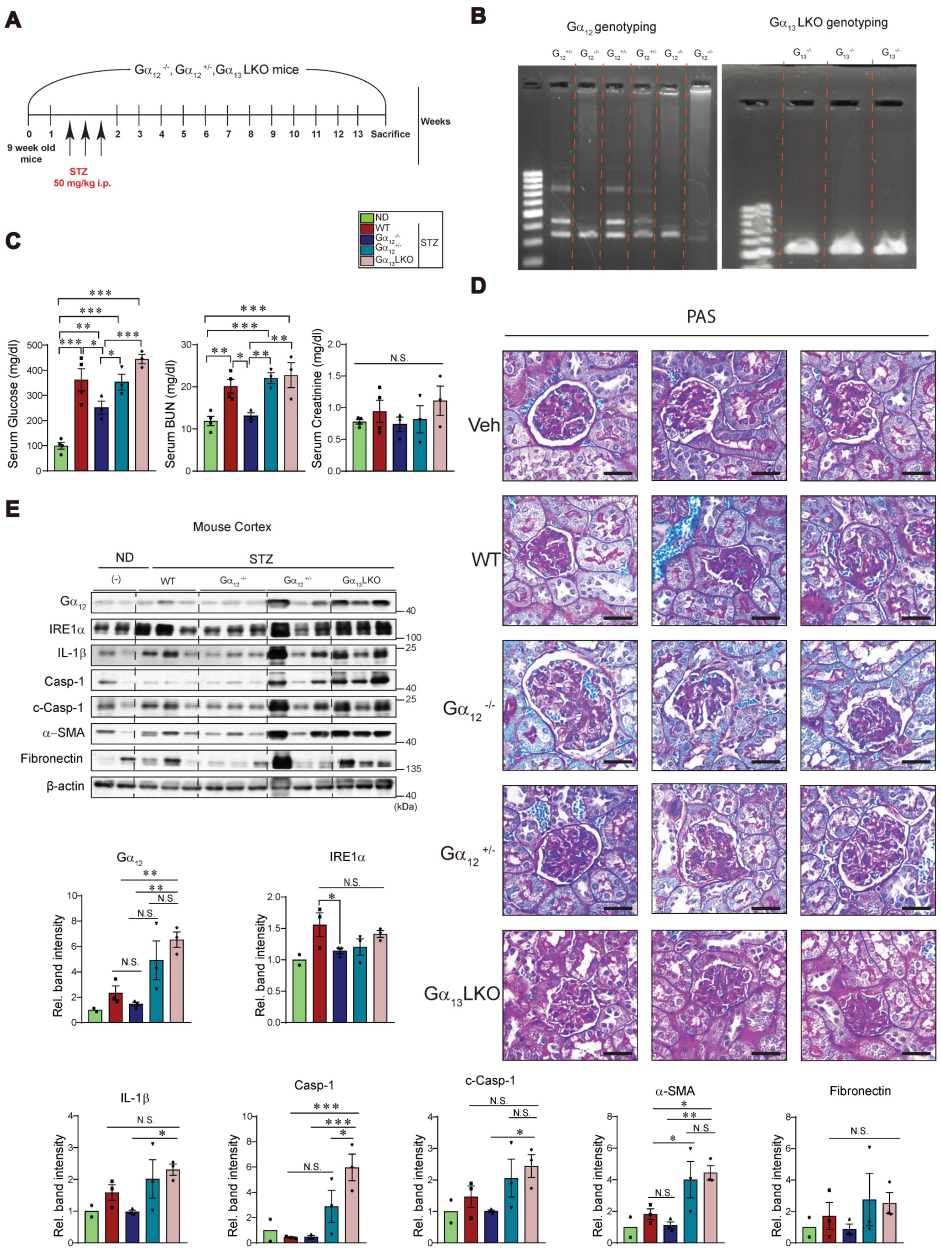
** Effects of Gα_12_ modulation on ER stress, pyroptosis, and fibrosis markers levels in WT, Gα_12_ KO, and Gα_13_LKO mice treated with STZ. (A)** A** s**chematic of the knockout (KO) animal study. Wild-type (WT), Gα_12_KO, heterozygous Gα_12_KO and Gα_13_ hepatocyte-specific knockout (Gα_13_LKO) mice were used to induce hyperglycemia (a 13-week STZ model). **(B)** Genotyping of the KO mice.** (C)** Serum RBS, CRE, and BUN contents (n = 4 for ND and WT; n = 3/each KO group). **(D)** PAS staining of kidney morphologies of the samples. Shown above are 3 different visual fields of one representative animal per each group (n = 4 for ND and WT; n = 3/each KO group). **(E)** Immunodetection assays for Gα_12_, IRE1α, mature IL-1β, total Casp-1, c-Casp-1, α-SMA, and fibronectin using the kidney cortex homogenates from the knockout (KO) animals subjected to STZ treatments to induce hyperglycemia (n=2 for ND, n = 3/each of the other groups). Values are represented as mean ± SEM (*p < 0.05, **p < 0.01, ***p < 0.001, and N.S., not significant). One-way ANOVA in association with an LSD multiple comparison was used for calculating statistical significance (**E**).

Finally, we were tempted to assess the effects of genetic modulation of Gα_12_ on our targets of interest using animals subjected to STZ treatment (a 13-week mouse model) to assess the minimal sufficient role of Gα_12_ in initiating injury pathways even in the absence of exogenous lipid overload (Figure 10A). This allowed us to specifically isolate the contribution of Gα_12_ driven solely by the metabolic derangement of hyperglycemia. Based on our previous research 7, 11, we used homozygous and heterozygous Gα_12_ KO animals, as well as Gα_13_ hepatocyte-specific KO (Gα_13_LKO) animals. In this set of experiments, we employed a STZ model instead of the HFD+STZ model to evaluate the possible enhancing effect by Gα_12_ overexpression as well as the decreasing effect by Gα_12_ ablation. After obtaining the KO animals, we performed a DNA analysis to verify their genotype using PCR and make the groups of either homozygous (Gα_12_-/-) or heterozygous (Gα_12_+/-) for the targeted gene (Figure 10B, left). Previously, Gα_13_ muscle-specific KO (Gα_13_MKO) animals exhibited a compensatory increase in Gα_12_ in skeletal muscle 16. Given this result, we were challenged to monitor the effect of Gα_13_LKO on Gα_12_ levels in the kidney and discovered an intriguing overexpression of Gα_12_ in these animals (Figure 10B, right). So, we additionally used the animals to evaluate Gα_12_ overexpression effect on hyperglycemia-induced ER stress, pyroptosis and fibrosis.

As expected, STZ treatment resulted in hyperglycemia and this effect was diminished by Gα_12_-/-, but not by Gα_12_+/- (Figure 10C, left). More interestingly, overexpression of Gα_12_ in the kidney as a consequence of Gα_13_LKO further enhanced hyperglycemia (Gα_12_-/- vs. Gα_13_LKO). We also found a significant decrease in blood BUN content in Gα_12_-/- animals as compared to WT animals (Figure 10C, middle). No change was found in serum creatinine contents (Figure 10C, right). Consistently, we were able to verify amelioration of renal histopathology by homozygous KO of Gα_12_ and histopathology aggravation by Gα_12_ overexpression (Figure 10D). Moreover, a homozygous deficiency of Gα_12_ prevented STZ-induced hyperglycemia from IRE1α induction (Figure 10E). Heterozygous KO (Gα_12_+/-), however, failed to do so, which may reflect a compensatory adaptation of the mice. In addition, overexpression of Gα_12_ in the kidney due to Gα_13_LKO markedly promoted pyroptosis and fibrosis in the kidney as compared to either WT or Gα_12_-/- mice. Conclusively, all of these results demonstrate that Gα_12_-dependent ER stress contributes to diabetic nephropathy and fibrosis complications by facilitating pyroptotic inflammasome pathways.

## Discussion

Diabetic kidney disease (DKD) is closely associated with abnormal renal tubular fatty acid metabolism, accelerating tubular injury and fibrosis 17. Thus, dysregulation of lipid homeostasis may contribute to the pathogenesis of diabetic kidney disease (DKD). Previously, we reported that severe ER stress leads to overexpression of Gα_12_ in hepatocytes 7, 13. In the present study, HFD feeding in combination with hyperglycemia upsurged ER stress accompanying overexpression of Gα_12_ in the kidney. The present study investigated the impact of Gα_12_ overexpression by which chronic hyperglycemia, exacerbated by excess FFAs, contributes to renal injury. Our findings first reveal that a combined HFD and STZ treatment in mice, a model designed to induce chronic hyperglycemia and liver steatosis, promotes more severe renal damage, as mediated through the processes including Gα_12_ overexpression, ER stress, pyroptosis, and fibrosis. The HFD and STZ combinatorial treatment caused the pronounced levels of IRE1 and ATF6, indicative of ER stress referring to the role of these events for renal complications 18.

Importantly, our results demonstrate for the first time the impact of ER stress accompanying Gα_12_ overexpression on the cellular stress responses. The pathological role of uncompensated ER stress showing no increase in GRP78 level is characterized by the increased expression of Gα_12_ shown herein, and the inability of ER to cope with ER stress 19, 20. ER stress also triggers the deubiquitinating USP14, enhancing CREB stability to aggravate glucagon action and gluconeogenesis 21. Moreover, it is likely that chronic exposure to hyperglycemia and FFA overload disrupts ER homeostasis. This contention is in line with the observation that FFAs stimulate ATF6-associated maladaptive response and aggravate insulin resistance 22. During ER stress, the ER repositions itself towards the perinuclear region of mitochondria, leading to enhanced coupling between the two organelles 23. This coupling then may promote super-complex formation of mitochondrial respiratory chain, oxidative phosphorylation activity and calcium transfer, leading to mitochondrial dysfunction 24.

ER-stress sensors may cause the overexpression of Ga_12_ via IRE1a-XBP1 axis 7, 25. It has been shown that the cargo-recruiting subunit of the coatomer protein II acts as a guanine nucleotide exchange factor to activate the signaling protein Gα_12_ at the ER exit sites, coordinating the ER export and preventing fluctuations of folded active cargo 26. As Gα_12_ is located 40% endogenously on mitochondria as well as ER 27, 28, an enhanced contact between the two organelles due to uncontrolled UPR could affect diabetic complications 7. Additionally, a prolonged and enhanced levels of FFAs would commutatively contribute to ER hemostasis imbalance 29, leading to more severe ER stress 30, which may be featured by the overexpression of Gα_12_. Hence, in the progression of diabetic pathogenesis, ER stress with Gα_12_ overexpression may facilitate kidney complications. This contention is strengthened by the present results of RNA seq dataset analysis of GSEA and Ontology analysis, Sankey diagram HALLMARK and WikiPathways obtained from the kidney of HFD+STZ-treated mice.

The Gα_12_ axis is involved in the stabilization of SIRT1, regulating mitochondrial (mt) oxidative respiration 16. The concurrent increase in PGC1α and PPARα expression suggests mitochondrial adaptive changes in mt-biogenesis, representing a compensatory response to metabolic stress. PPARβ is involved in the activation of PPARα via NRF-1 activation and contributes to mt-biogenesis. Studies have proven that reduced expression of PPARα attenuates the increased mt-biogenesis 31. ER stress promotes obesity-associated insulin resistance by increasing expressions connexin 43 which promotes ER stress to neighboring cells 32. Thus, chronic situation of hyperglycemia and lipid overload would aggravate the production of ROS in mitochondria 33.

The Gα_12_ axis additionally plays a role in cellular processes such as necroptosis and the accompanying inflammation in the APAP-intoxicated liver 7. Our studies using genetic models or human specimens showed key molecules including specific microRNAs responsible for necroptic liver injury. Pyroptosis, another form of cell death, also contributes to inflammatory tissue damage. While the UPR initially aims to restore ER function, prolonged severe stress may trigger cell death pathways including pyroptosis. Our results shown here demonstrate for the first time that sustained ER stress accompanying Gα_12_ overexpression contributes to pyroptosis, worsening diabetic kidney complications. In the kidney of HFD+STZ-treated mice, our results demonstrated robust pyroptosis, characterized by NLRP3 and IL-1β activation in the kidney, being in line with the observation that inhibition of IRE1a RNase inhibited activation of NLRP3 and pro-IL1β 34. The positive correlations between Gα_12_ expression and pyroptosis markers also strengthen the link between Gα_12_ signaling and inflammatory cell death. IRE1α activation, which may lead to upsurged ROS levels via inhibition of microRNA-17 and by enhancing *Txnip* mRNA stability 35, may lead to increase in Gα_12_ level. These events would promote NLRP3 translocation and pyroptosis activation 35, also enhancing IRE1 activation and IRE1-mediated transcriptional induction of Gα_12_ 7.

In the present research, Kim-1 levels were moderately increased only in the medulla, but not in the cortex, suggesting regional vulnerability of kidney tubules by HFD+STZ. This idea is supported in part by the observation of Bonventre et al. study that Kim-1 was elevated in proximal tubules as well as in the urine of diabetic humans 36. Briefly, Kim-1 facilitates PA-bound albumin uptake, resulting in enhanced tubular injury, whereas this effect was ameliorated by genetic ablation of Kim-1 in HFD and STZ DKD model 36. Our results shown in the present study support the concept that hyperglycemia plus fat overload may have a major impact on glomeruli, but moderately affect tubular cells. Further studies are needed to understand the basis underlying this regional difference. In our study, ultrastructural analyses confirmed the detrimental effects of hyperglycemia plus fat overload on kidney. Accumulation of large vacuoles, tubular degeneration, podocyte foot process effacement, and mitochondrial swelling and fragmentation with disrupted cristae confirmed mitochondrial changes in HFD+STZ-treated mice. This finding is consistent with the observation that diabetic kidney exhibits diffused thickening of glomerular basement membrane and fused foot processes within the glomeruli 37. Activation of cell death process due to chronic ER stress results in loss of podocytes, mature differentiated cells with lack of regeneration capacity 38. Podocytopenia is also found due to cytoskeletal remodeling 39, triggering fibrogenic pathways 40. Our findings on podocyte loss and podocytopenia with altered basement structure may be due to circulating FFAs which would promote ER stress 41, 42. Together, our findings provide evidence that sustained hyperglycemia with fat overload induces renal injury and the consequent diabetic complications.

Fibrosis, featured by excessive ECM accumulation, leads to progressive scarring and further loss of renal function 43. In particular, diabetic patients may experience interstitial fibrosis 44, 45. In the present study, we found notable increases in fibrosis markers in both renal cortex and medulla by HFD+STZ. Our results also verified a pronounced increase in fibronectin levels, particularly in the cortex, suggesting its potential as an early and sensitive biomarker for renal fibrosis. This contention is fortified by our finding that HG+PA treatment causes the induction of fibronectin in HEK293 cells as early as 1.5 h after treatment. Microvascular occlusion causing exaggerated mitochondrial ROS, inhibition of mitochondrial fission and mt-DNA depletion may account for the ECM accumulation, also promoting upsurged inflammatory responses and EMT 46. In another study, loss of podocytes due to the deletion of SHP-1 causes accumulation of fibronectin as well as collagen IV 44.

In contrast to the notable kidney complications in mice exposed to HFD+STZ, our study into STZ-induced hyperglycemia revealed an absence of severe renal pathology. Despite a substantial and a distinct increase in blood glucose levels (i.e., 440 mg/dl in STZ-treated vs. 250 mg/dl in HFD+STZ-treated mice), the kidneys of STZ-alone mice exhibited a relatively benign response. An assessment of pyroptosis markers also showed no significant changes in both pro-caspase-1 and active caspase-1 levels. However, we could see a two-fold increase in IL-1β level, which was less pronounced than that caused by HFD+STZ. This limited inflammasome response supports the notion that hyperglycemia factor alone does not induce a full-blown inflammasome cascade, as also supported by the lack of increase in the CRE content. Consistently, we observed no notable changes in fibrosis markers, as corroborated by histopathological analyses and glomeruli/Bowman's capsule ratios. Crucially, we found no significant alterations in ER stress markers or Gα_12_ within the kidney, suggesting that hyperglycemia alone may not be a sufficient trigger for ER stress-dependent renal complications. Our results highlight the complex interplay of factors in DKD pathogenesis, suggesting that hyperglycemia may synergize with other metabolic insults to manifest renal complications.

Further, we investigated the combined effect of HG and FFA on cellular fate using HEK293 and HK-2 cells to assess their roles in pyroptosis and fibrosis. As expected, prolonged exposure of the cells to HG and PA (i.e., 8-48 h) enhanced cell death, emphasizing the detrimental impact. Treatment with PA alone or in combination with HG for 1.5 or 3 h induced both IRE1 and Gα_12_, displaying a synergistic or additive effect on Casp-1 and IL-1β. Immunodetection assays provided further evidence of increased α-SMA and fibronectin intensities, linking the combined metabolic insult to pro-fibrotic changes. These results reinforce that the coexistence of HG and FFAs promotes both pyroptosis and fibrosis, providing a cellular explanation as to how fat overload exacerbates kidney complications. Another intriguing observation of this study is that genetic overexpression of Gα_12_ augmented the detrimental effect of HG and FFA on pyroptosis and fibrogenesis, while its siRNA knockdown prevented it. Moreover, our *in vivo* results using genetic KO models verified the *bona fide* impact of ablation of Gα_12_ and overexpression of Gα_12_ as a consequence of Gα_13_LKO on pyroptosis and fibrogenesis, accounting for the novel mechanistic basis underlying renal complications.

Another interesting observation is the amelioration of the pathological changes by AZ2, a small-molecule inhibitor targeting the Gα_12_ signaling pathway, highlighting its therapeutic utility in diabetic nephropathy. AZ2 reversed pyroptotic changes, indicating its potential to suppress inflammation and treat complications. AZ2 also had the capability to restore podocyte structure and reverse mitochondrial changes. Additionally, this agent inhibited the expression of fibrosis markers. In prior studies, we demonstrated that pharmacological inhibitor of Gα_12_ using AZ2 effectively ameliorated hyperglycemia and restored metabolic homeostasis across multiple diabetic animal models 13. Furthermore, independent research from our group expanded the potential clinical utility of this compound, revealing that AZ2 treatment reduced disease severity and inflammation in murine models of inflammatory bowel disease 9. Collectively, these findings support the role of Gα_12_ signaling in metabolic and inflammatory pathologies. The compound, for which pharmaceutical formulation is being optimized, is currently in preclinical stage. While our results support its mechanistic efficacy, safety and toxicity evaluations are further required.

In conclusion, our research indicates that sustained hyperglycemia, particularly when worsened by FFA, is a critical driver of renal injury. This damage occurs through a cascade of events involving the overexpression of Gα_12_, leading to severe ER stress, pyroptosis, and fibrosis. This study also identifies AZ2 as a promising candidate, demonstrating its effectiveness in renal pathological changes. Future studies should focus on fully elucidating the precise mechanisms by which AZ2 exerts its pharmacological effect. In addition, our analysis of human RNA-seq dataset verified a cluster of genes responsible for the signal transduction pathway for NLRP3 activation, inflammatory response, immune response and cell adhesion in patients with DKD. Hence, translational studies will ultimately be vital to determine the efficacy and safety of AZ2 in patients.

## Supplementary Material

Supplementary figures.

## Abbreviations

α-SMA: alpha-Smooth Muscle Actin
ANOVA: Analysis of Variance
ATF6: Activating Transcription Factor 6
BSA: Bovine Serum Albumin
BUN: Blood Urea Nitrogen
ciGEnC: conditionally immortalized human glomerular endothelial cell
CRE: Creatinine
DKD: Diabetic Kidney Disease
ECL: Enhanced Chemiluminescence
ECM: Extracellular Matrix
EMT: Epithelial-Mesenchymal Transition
ER: Endoplasmic Reticulum
FFAs: Free Fatty Acids
PAS: Periodic Acid-Schiff Staining
Gα_12_-/-: Homozygous Knockout of Gα_12_
Gα_12_+/-: Heterozygous Knockout of Gα_12_
Gα_13_LKO: Liver-Specific Knockout of Gα_13_
HFD: High Fat Diet
HG: High Glucose
HRP: Horseradish Peroxidase
i.p.: intraperitoneal
IACUC: Institutional Animal Care and Use Committee
IL-1β: Interleukin-1β
IRE1α: Inositol-Requiring Enzyme 1α
LG: Low Glucose
LSD: Least Significant Difference
ND: Normal Diet
NLRP3: NLR Family Pyrin Domain Containing 3
PA: Palmitic Acid
PBS: Phosphate-Buffered Saline
PGC1α: Peroxisome Proliferator-Activated Receptor Gamma Coactivator-1α
p.o.: per os (oral administration)
PPARα: Peroxisome Proliferator-Activated Receptor α
ROS: Reactive Oxygen Species
SDS-PAGE: Sodium Dodecyl Sulfate-Polyacrylamide Gel Electrophoresis
STZ: Streptozotocin
TBST: Tris-Buffered Saline with Tween 20
TEM: Transmission Electron Microscopy
UPR: Unfolded Protein Response
VEH: Vehicle
