# Supplementary material for: Hyperglycemia in combination with excess fat intake promotes renal pyroptosis and fibrosis through Gα12-dependent endoplasmic reticulum stress
Source: Theranostics. 2026 Jan 14;16(8):3996–4018. doi: 10.7150/thno.124015 (PMC12905788; doi:10.7150/thno.124015)
Supplement: Supplementary file 1 — Supplementary figures. [file thnov16p3996s1.pdf]

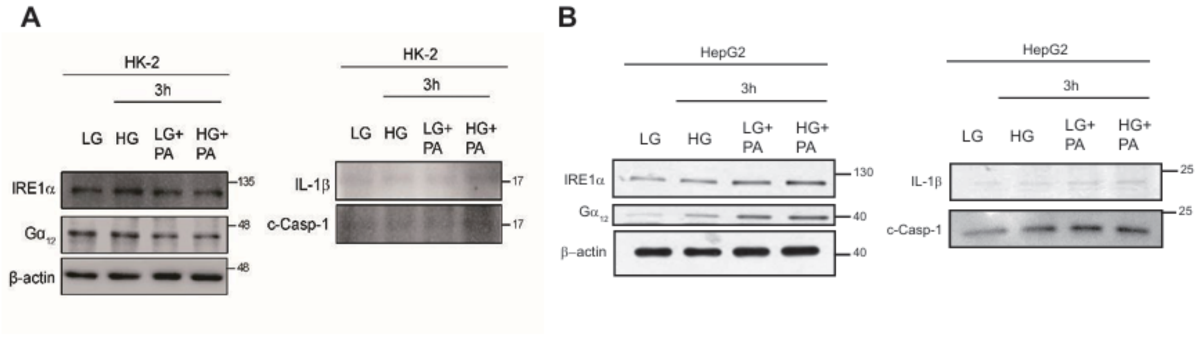

Supplementary Figure 1. Effects of HG and/or PA treatments on ER stress and pyroptosis markers in HK-2 **(A)** or HepG2 cells **(B)**.

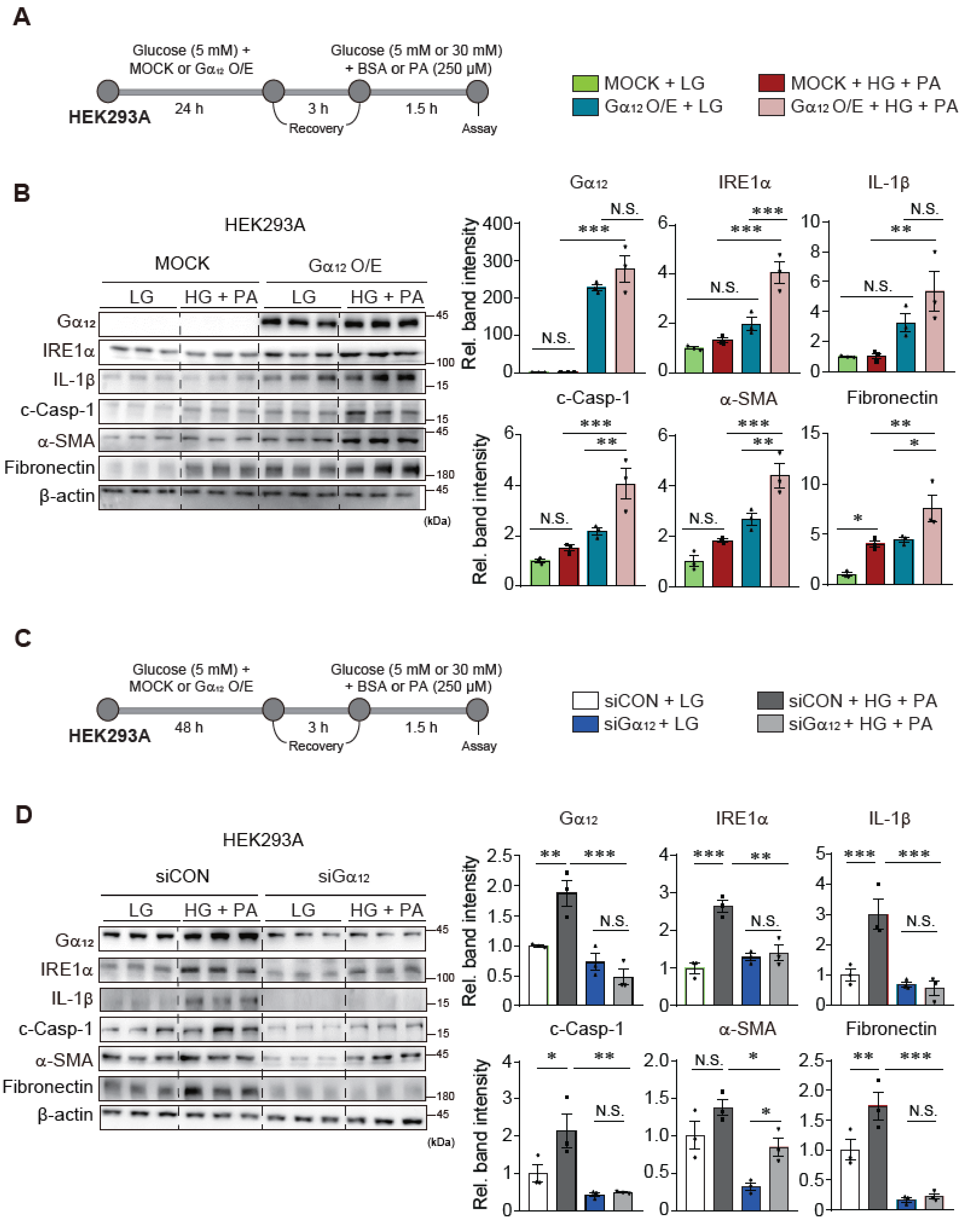

Supplementary Figure 2. Effect of Gα12 overexpression or knockdown on ER stress-, pyroptosis-, and fibrosis-associated proteins in HEK293A cells treated with HG plus PA

(A) Schematic illustration of Gα_{12} overexpression experiment in HEK293A cells. **(B)** Immunoblots of Gα_{12}, IRE1α, IL-1β, c-Casp, fibronectin and α-SMA in HEK293A cells transfected with a Gα_{12}-encoding plasmid or MOCK, and subsequently exposed to high glucose and palmitic acid (HG+PA) (n = 3 each). **(C)** Experimental scheme depicting Gα_{12} siRNA knockdown experiment in HEK293A cells. **(D)** Immunodetection assays in the cells treated with HG+PA after transfection with siRNA targeting Gα_{12} or control siRNA (siCon) (n = 3 each). Values are represented as mean ± SEM (*$p < 0.05$, **$p < 0.01$, ***$p < 0.001$, and N.S., not significant). One-way ANOVA in association with a Tukey's multiple comparison was done to assess statistical significance (**B, D**).

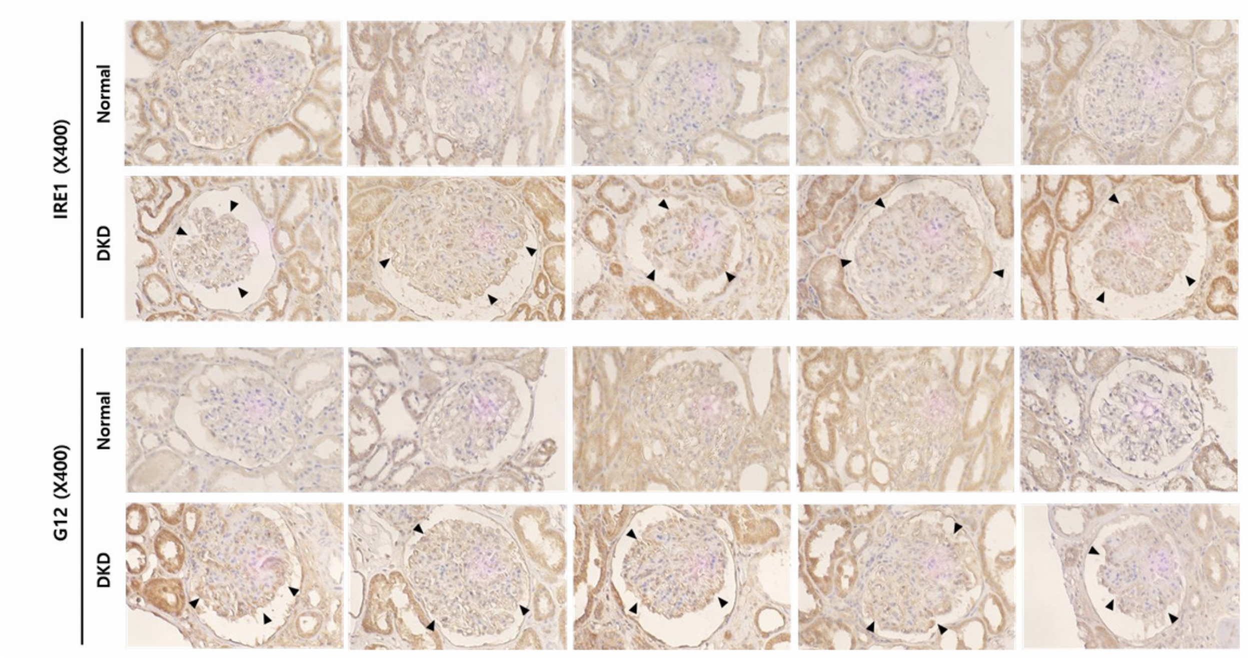

Supplementary Figure 3. Immunostaining of human kidney morphologies. Shown above are representative images from the tissue samples of normal individuals and DKD patients (n = 4 for normal vs. n = 10 for DKD). Arrowheads indicate the regions of either IRE1 or Gα_{12} overexpression.
